# Obtaining a PhD in Portugal. Determinants of Success and Subsequent Career Paths

**DOI:** 10.12688/f1000research.150552.1

**Published:** 2024-09-27

**Authors:** Ana Ramos, Daniel Ferreira

**Affiliations:** 1 Studies and Planning Division, Foundation for Science and Technology, Lisbon, Portugal

**Keywords:** PhD success rate, Time to degree, Doctorate holders, Career paths

## Abstract

**Background:**

Over the past three decades, Portugal has invested significantly in doctoral training. However, there is a lack of up-to-date information on the success of scholarship holders in obtaining the degree or on their subsequent career paths.

**Methods:**

This study analysed four cohorts of scholarship holders funded by the Foundation for Science and Technology between 1995 and 2012. The professional situation at different points in time after the Ph.D. (5, 10, 15 and 20 years) was studied.

**Results:**

The success rate for obtaining the degree was 88%, which is comparable to other European countries for doctorates completed with funding over a period of 3-4 years. The average time to obtain the degree was 5.06 years. The time to degree was influenced by the scientific field, the nationality of the scholarship holder, and the location of the institution awarding the degree (in Portugal or abroad). The gender or age range of the scholarship recipients did not influence the time required for graduation.

The analysis of the career and sector of activity of these graduates at different points in time after obtaining the Ph.D. revealed that approximately 60% percent of the graduates were engaged in R&D activities at some point in their career paths, but this percentage diminished from the oldest to the most recent cohorts, suggesting difficulties in retaining recent graduates in academia and in the private sector. In 2020, 63% of the graduates were engaged in R&D activities, and almost half were employed in higher education as teachers, researchers, or scholarship holders.

**Conclusions:**

This study confirmed the challenges faced by doctoral graduates in integrating into non-academic job markets. On the other hand, it has demonstrated an efficient use of public funds, with high success rates and a time to degree that is within the average of other European countries.

## Introduction

### Doctoral training in Portugal – background

In Portugal, public support for training human resources in science and technology began in 1987 with the launch of the Science and Technology Mobilising Programme (Programa Mobilizador de Ciência e Tecnologia, PMCT). The programme funded master or doctoral scholarships with the aim to address the shortage of human resources in science and technology and complement the traditional way of recruiting research assistants in higher education institutions and state laboratories ( Rodrigues, 2017). Since then, the attribution of doctoral scholarships (3-4 years duration, in Portugal or abroad) has been at the core of the activities of the main funding agencies: the *Junta Nacional de Investigação Científica e Tecnológica* (JNICT) until 1996 and the *Fundação para a Ciência e a Tecnologia* (FCT) since 1997. The FCT has supported over 30000 Ph.D. candidates through various mechanisms, including annual calls, doctoral programmes or international partnerships. Over the course of 25 years, the FCT has invested more than 3.2 billion euros in master, doctoral, and postdoctoral scholarships. Horta et al. (2018) confirmed the positive effects of the FCT funding on doctoral training, providing stability for research and leading to the production and consolidation of knowledge necessary for thesis writing. The clear focus on research objectives also contributed to the scientific development of fields, institutions, and scientific systems, an effect observed both during and after doctoral studies.

The investment has led to a significant increase in the number of doctorates awarded in Portugal; its cumulative number was almost 38000 in 2022 ( Direção-Geral de Estatísticas de Educação e Ciência [DGEEC], 2023). This investment is also evident in the number of doctoral graduates residing in Portugal, which increased from 19034 in 2009 to 30807 in 2015 and 37113 in 2020, corresponding to a doubling in about a decade ( DGEEC 2013, 2017, 2021).

Despite the significant growth in the number of degrees awarded and of doctorate holders living in Portugal, in 2022 the proportion of doctorate holders in the labour force (25-64 years old) was 0.9%, still lower than the average for OECD countries, 1.3% ( Organization for Economic Cooperation and Development [OECD], 2023). However, the new doctoral graduates per 1000 inhabitants in the 25-34 age group has been rising. In 2015, Portugal “produced” 1.9 new doctorates per 1000 inhabitants aged 25-34 years, which was similar to Spain’s rate of 1.91 and close to the EU average of 2.01 ( European Commission [EC], 2017). This led to a substantial increase in the number of new doctorate holders in recent years.

The increased number of doctorate holders has presented significant challenges for their professional integration, particularly for younger individuals, both within the higher education sector, as well as in non-academic sectors such as businesses or not-for-profit organisations. In the higher education sector, access to teaching positions is limited, as the number of academic staff has remained stable for the past 20 years. Because of this shortage of positions, there is an imbalance between supply and demand, resulting in a significant number of Ph.D. graduates competing for scarce job opportunities or, more often, postdoctoral fellowships ( OECD, 2019). From 1994 to 2016, FCT and its predecessor JNICT awarded over 10000 postdoctoral fellowships for a period of up to six years with a mid-term evaluation after three years. From 2007 onwards, programmes were introduced to provide more stable conditions for postdoctoral researchers. Until 2015, these programmes offered 5-years fixed-term contracts with no perspective of further career development. In 2016, the government launched a new initiative (the Scientific Employment Stimulus Programme) to promote more stability and better social protection for Ph.D. holders by providing them with salaried positions with 6-years employment contracts. The underlying legal regime obliges the public higher education and research institutions to open a competitive recruitment procedure to provide an employment opportunity to these researchers at the end of the 6-year contract.

### Career paths of doctoral graduates

There is currently limited understanding of the expansion of doctoral training, including its causes, social and economic contributions, and medium to long-term consequences. Although there is a broad consensus on the social and economic importance of doctorates, data collection at the international level is inconsistent and the available information is primarily based on ‘snapshot’ surveys. Only recently have some countries, such as the USA, Germany, and the Netherlands, established large-scale surveys that will enable longitudinal analyses of the career paths of doctoral graduates ( Hancock et al., 2019). The results of these studies will complement previous analyses carried out by the OECD using the CDH survey or in the context of the MORE projects funded by the European Commission.

Doctoral degree holders have lower unemployment rates than graduates of other educational levels and receive a significant wage premium ( Auriol et al., 2013; Pedersen, 2014). It has been revealed that there is an increasing tendency for doctoral graduates to find placements outside academia, although their employment paths and the distribution by sector varies considerably between countries ( Auriol et al., 2013). The ‘DocEnhance’ study, funded by the Horizon 2020 programme, surveyed 2200 individuals with a doctorate earned from European universities between 2016 and 2020. The results showed that doctoral graduates find jobs quickly after graduating, both inside and outside academia. The unemployment rate was very low, at 3%, which is lower than the average for the European Union labour force (7%). The academic sector, including universities and research institutes, was the most significant employment sector. Temporary contracts accounted for approximately 25%, which is more than double of the EU rate ( Boman et al., 2021). In the UK, almost 30% of individuals worked in academia 3.5 years after obtaining their Ph.D. Of these, 70% were teaching professionals and 30% were university researchers. Around 20% worked in industry, often as researchers or managers. Another 20% held medical jobs, including as practitioners and medical scientists ( Hancock, 2021). The ‘10,000 PhDs’ project at the University of Toronto in Canada revealed that doctoral degrees led to a wide range of positions. Approximately 23% of the respondents held tenure or tenure-track positions, while slightly over half worked in academic positions, including administrative roles. Nearly 30% worked in the industry, and others were employed by federal or provincial governments, charities, or entrepreneurial businesses ( Woolston, 2018). All the studies mentioned above used surveys and therefore provide limited perspectives due to low response rates and difficulties in locating individuals to be surveyed. The Centre for Research and Development Monitoring in Flanders (ECOOM) used a database containing complete records of academic staff, doctoral students, and degrees awarded by the five Flemish universities to investigate the factors that contribute to obtaining a doctorate and subsequent career paths ( Groenvynck et al., 2013; Debacker, 2021). These are some of the few studies that used administrative data to study career paths.

Upstream of professional destinations and employability, completion rates and time to degree are important indicators for monitoring the stock and flow of researchers on the labour market and for assessing the efficiency and effectiveness of doctoral training ( Wollast et al., 2018; Groenvynck et al., 2013). Prolonged time-to-degree and high drop-out rates have negative effects on all involved parties: Ph.D. candidates, Ph.D. organisers or supervisors, and funding bodies. In some studies, the completion rate has been estimated at 50%. However, research has shown that there is a strong positive correlation between the success rate and the availability of funding for an adequate period, which allows candidates to dedicate themselves exclusively to their studies ( Ehrenberg & Mavros, 1995; Groenvynck et al., 2013).

Although Portugal has invested considerably in doctoral training over the last three decades, there is a lack of up-to-date information on the success of scholarship holders in obtaining a doctoral degree or on the subsequent career paths. The CDH survey, mentioned above, provides valuable information on the ‘pool’ of doctoral graduates living in Portugal in the survey years (approximately every 5 years), but does not provide information on their career paths. The latest CDH survey figures (2020) reveal a low unemployment rate (2.3%) among doctorate holders and a high prevalence of the Higher Education sector in their occupation (77%), followed by the State (13%), Businesses (8%), and Private Non-Profit Institutions (2%) sectors. Over 80% of the doctoral graduates residing in our country were engaged in R&D activities, with 68% having a permanent contract and 32% having a fixed-term contract. Recent publications on doctoral graduates in our country have focused on specific groups and narrower themes. For example, Morais and Gaio Alves (2019) studied graduates from a particular university, while Spognardi and Matos (2021) and Ferreira (2023) examined the precariousness of the labour situation. However, these studies do not provide specific information on individuals who have benefited from an FCT grant.

To bring a comprehensive perspective on doctoral graduates funded by the FCT, this study analysed the career paths of 5800 individuals who benefited from a doctoral scholarship, using four cohorts of scholarships which began in 1995-1997, 2001-2002, 2006, and 2012. This study is particularly timely in the context of European and national policies, where attractive and sustainable research careers have been recognised as strategic. The results can help to establish indicators for monitoring the policies implemented over the last 30 years, as well as for defining future actions and policies. The aim was to clarify the following questions:

1. How do different groups of scholarship recipients compare in terms of success rate and time to obtain a degree, based on variables such as gender, nationality, age at the start of the scholarship, field of study, and location of the awarding university?
2. What is the persistence of these doctoral graduates in the Portuguese R&D system and what are the characteristics of their post-doctoral careers?
3. What are the factors that explain career paths and what conclusions are relevant for public policy?

## Methods

### Rationale for cohort’s definition

Based on literature data suggesting that the average time required for concluding a Ph.D. is 5 years, four cohorts of FCT scholarship holders were defined: cohort 1 included Ph.D. scholarships started between 1995 and 1997, cohort 2 covered scholarships started in 2001-2002, and cohorts 3 and 4 consisted of scholarships started in 2006 and 2012, respectively. The grant recipients from four cohorts, totaling 5819 individuals (see Table 1), were studied to determine their labour situation at various points in time. The initial assumption was that the scholarship recipients who began their studies between 1995 and 1997 would have completed their degrees mostly between 1999 and 2001. This would allow for the determination of their professional situation approximately 5 years later (2004-2006), 10 years later (2009-2011), 15 years later (2014-2016), and 20 years later (2019-2021). For scholarships beginning in 2001-2002, the professional situation would be evaluated at 5 years (2009-2010), 10 years (2014-2015), and 15 years (2019-2020) after obtaining the degrees. For the 2006 cohort, who obtained their degree in 2011, their situation would be assessed 5 years later in 2014, and again 10 years later in 2019. The 2012 cohort’s labour situation would be appraised in 2020, approximately 5 years after obtaining their degree.

**Table 1.** Cohort definition.

Cohort number	Year of scholarship	Estimated degree year+5	Estimated degree year+10	Estimated degree year+15	Estimated degree year+20
**1**	1995+1996+1997	2004-2006	2009-2011	2014-2016	2019-2021
**2**	2001+2002	2009-2010	2014-2015	2019-2020	
**3**	2006	2014	2019		
**4**	2012	2020			

Rationale for cohort definition. Cohort 1 included Ph.D. scholarships started between 1995 and 1997, cohort 2 covered scholarships started in 2001-2002, and cohorts 3 and 4 consisted of scholarships started in 2006 and 2012, respectively. Assuming that on average it takes 5 years to obtain a Ph.D., scholarship recipients who began their studies between 1995 and 1997 would have completed their degrees mostly between 1999 and 2001. This would allow for the determination of their professional situation approximately 5 years later (2004-2006), 10 years later (2009-2011), 15 years later (2014-2016), and 20 years later (2019-2021). The same rationale applies to the remaining cohorts.

### Determination of the graduation year

To determine the year of graduation, various sources of information were consulted, including the FCT databases, the National Register of Doctoral Theses (RENATES), the Ciência ID ^1^ identifier (when available in the FCT databases) via the Ciência Vitae ^2^ platform, as well as the physical individual files of scholars available at the Science and Technology Archive of the FCT. When information could not be obtained through the aforementioned sources, we used the scholarship holder’s name and the institution that awarded the degree to search national and international bibliographic repositories, research units’ and higher education institutions’ websites, LinkedIn, etc. Due to the incompleteness of information about the graduation year in administrative databases, success rate in this paper refers to individuals for whom it was possible to determine the year of graduation using the approach described above and the sources listed (up to July 2022).

### Professional status after graduation

To ascertain the professional status of past scholarship recipients at different points in time following their doctoral degree, we used two distinct sources of data: the Portuguese R&D Survey (hereafter IPCTN) and the Careers of Doctorate Holders (CDH) survey. The IPCTN is an annual survey that provides official statistical information on the human and financial resources allocated to R&D activities in Portugal. It collects information on the individuals engaged in R&D activities, including their career (teaching, research, medical, etc.), category (assistant professor, principal investigator, etc.), and the sector they are associated with (Business, State, Higher Education, or Private Non-Profit Institution). The CDH is a survey aimed at doctoral graduates residing in Portugal, with or without R&D activity; its latest edition was conducted in 2020.

For individuals whose graduation year (N) was known, their data (name, date of birth and ID number) were cross-referenced with the IPCTN data. Professional situations were assigned to those located at N+5 years, N+10 years, N+15 years, and N+20 years, including their career, category, and sector of activity. The professional situation in 2020, the last year consulted for the IPCTN, was also assigned, regardless of the time elapsed since graduation. When an individual worked in R&D in two or more organisations in the same year, each situation had to be analysed on a case-by-case basis in order to assign the doctorate to the career/category/activity sector that best reflected his or her professional situation in that year.

The data of those individuals whose graduation year was known was also cross-referenced with the data collected in the CDH survey for 2020. This complemented the IPCTN data and characterised the professional situation in 2020, regardless of the year of the degree or the time elapsed, providing a snapshot for that year. The aim was to locate as many former doctoral students as possible, understand their career paths and determine their employment situation in 2020.

The results of all cross-checks were anonymised and processed in a safe center. Their disclosure follows the principles of statistical confidentiality and personal data protection.

## Results

### Cohort characterization

The demographic characteristics of the Ph.D. candidates and the distribution of scholarships by scientific field and by location of the degree awarding institution are shown in Table 2. The comparison of cohort 1 with cohort 4 reveals that the proportion of women increased from 46.5% to 57.7%, most likely due to the mounting predominance of female graduates in secondary and tertiary education. Foreign scholarship holders (mostly from Brazil and Italy) expanded from 2.5 to 8.7%. The percentage of Ph.D. candidates aged 35 or over at the application date rose from 8.3% to 14.5%. The drop in the success rate of FCT doctoral scholarship calls, from 2009 onwards, may have contributed to this, since candidates who were not funded in a given call tried again in subsequent year(s) and, consequently, the age of successful candidates tended to rise.

**Table 2.** Cohort characterization.

Cohort	1	2	3	4
	N=1122	N=1196	N=1944	N=1557
Year of scholarship	1995+96+97	2001+02	2006	2012
% Women	46.5%	54.4%	55.6%	57.7%
% Age ≥ 35 ^1^	8.3%	9,9%	13.8%	14.5%
% Foreign	2.5%	6.0%	7.2%	8.7%
% Scholarships abroad	34.1%	24.1%	19.0%	11.9%
Scientific field ^2^				
Agricultural and veterinary sciences	5.1%	4.0%	3.2%	4.5%
Engineering and technology	30.0%	18.6%	21.9%	22.2%
Natural sciences	32.1%	39.6%	26.9%	19.5%
Medical and health sciences	12.7%	10.0%	10.8%	14.1%
Social sciences	13.6%	17.9%	26.1%	26.8%
Humanities and the arts	6.4%	9.9%	11.2%	12.9%

Demographic characteristics of the Ph.D. candidates and the distribution of scholarships by scientific field and by location of the degree awarding institution.

^1^ Age at application year.

^2^ FORD, Fields of Research & Development, OECD (2015), Frascati Manual 2015: Guidelines for Collecting and Reporting Data on Research and Experimental Development.

The number of scholarships awarded to degree-granting institutions outside Portugal fell significantly and steadily from cohort 1 to 4. In cohort 1, the 383 scholarships awarded for Ph.D. studies abroad corresponded to 37.9%, while in cohort 4 this percentage had fallen to 9.7%. In all cohorts, the United Kingdom and the USA were the main destination countries for FCT-funded doctoral students. Spain and France came third and fourth, followed by Germany and the Netherlands.

The Social Sciences and the Humanities and Arts accounted for around 40% of the scholarships of cohort 4, an increase of 20 p.p. from cohort 1. Concomitantly there was a decline of the same magnitude in the proportion of grants in the fields of Natural Sciences, Engineering and Technology.

### Descriptive analysis of doctoral success and time to degree

Out of the 5819 Ph.D. candidates studied, at least 5093 (87.5%) obtained their degree. The percentage ranged from 93.1% in cohort 2 to 81.2% in cohort 4 ( Table 3). However, it is possible that some individuals in this latter cohort were still completing their work at the time of this analysis (July 2022). The proportion of scholarship recipients who obtained their degree abroad declined steadily from the 1995-1997 cohort to the 2012 cohort, from 27.2% to 11.3%.

**Table 3.** Success rate and time to degree.

Cohort	Year of scholarship	Number of scholarships	Number of degrees awarded ^1^ (Success rate)	Number of degrees abroad (%)	Average Time to degree (Years) ^2^
1	1995+1996+1997	1122	1007 (89.8%)	274 (27.2%)	5.54
2	2001+2002	1196	1114 (93.1%)	260 (23.3%)	5.33
3	2006	1944	1708 (87.9%)	317 (18.6%)	4.87
4	2012	1557	1264 (81.2%)	143 (11.3%)	4.67
	**All cohorts**	**5819**	**5093 (87.5%)**	**994 (20.1%)**	**5.06**

Descriptive analysis of doctoral success and time to degree.

^1^ Degrees awarded refers to the number of individuals for whom it was possible to determine the year in which they obtained their degree from various sources of information (until July 2022).

^2^ Average number of years between the start of the scholarship and the defense of the thesis.

The time to degree (TTD) for the Ph.D. candidates in this study was calculated as the difference between the year of the thesis defense and the year of the beginning of the scholarship. The average TTD was 5.06 years (Standard Deviation=2.11) and the median was 5.00 years. It is important to note that all Ph.D. candidates for whom the year of the thesis defense could be determined were included in this calculation, regardless of the time elapsed since the start of the grant, which ranged from 1 to 25 years. Some methodological issues that may affect the estimation of the TTD should be mentioned: we do not know if the beginning of training corresponded to the start date of the scholarship, as there are situations in which these moments do not coincide (e.g. previous scholarships or other funding). Additionally, there was no data available on the time lag between thesis submission and degree award, which can be significant and may artificially inflate the TTD.

The TTD decreased consistently from 5.54 years in cohort 1 (scholarships started in 1995-1997) to 4.67 years in cohort 4 (scholarships started in 2012). It is noteworthy that the maximum number of years to obtain the degree steadily dropped from cohort 1 (25 years) to cohort 4 (10 years), although in the latter the maximum interval may be underestimated, given the possibility that there were Ph.D. candidates still completing their work at the time of analysis. The reason why some scholarship holders obtained their degree in less than three years may be attributed to the availability of prior funding. For instance, older cohorts may have received master scholarships, while more recent cohorts may have received funding from their host institutions.

This trend to reduced time to degree has also been observed in studies conducted in other European countries. One of the reasons is the increasing prominence of doctoral programmes, to the detriment of the “student-supervisor model”, as well as the implementation of the Bologna process. Currently, the awarding of doctoral degrees by Portuguese higher education institutions is subject to several conditions. These include the presence of a qualified teaching staff, primarily composed of individuals who hold doctoral degrees, as well as other human and material resources that ensure the quality of training. The fulfilment of these requirements is verified through an accreditation process conducted by the Higher Education Assessment and Accreditation Agency (A3ES), which was established in 2007.

**Figure 1.**
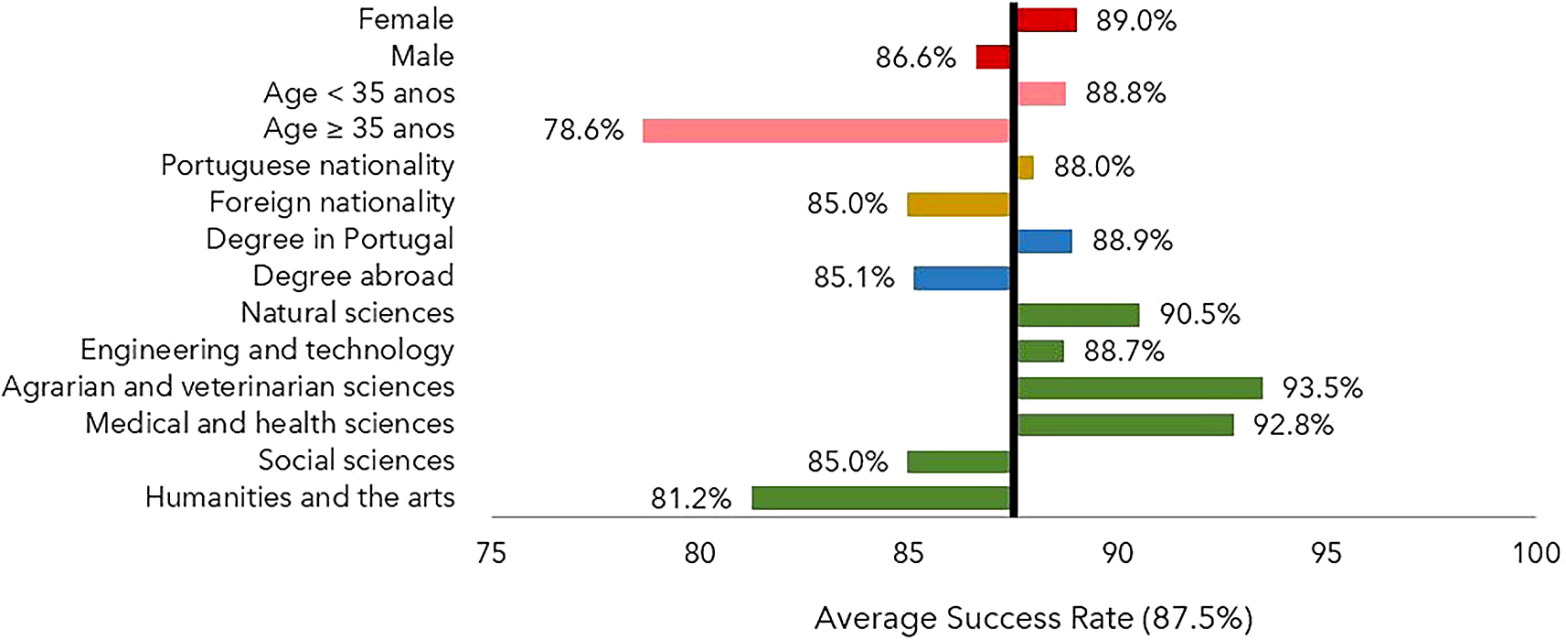
Success rate, all cohorts. The success rate is defined as the percentage of individuals who obtained the degree until July 2022 and is shown as a deviation from the average success rate, 87.5%. Notes: Age at application year. FORD Fields of Research & Development, OECD (2015), Frascati Manual 2015: Guidelines for Collecting and Reporting Data on Research and Experimental Development.

The percentage of individuals who obtained a degree was slightly higher for women than for men, as shown in Figure 1. Portuguese nationals achieved a moderately higher success rate than foreigners, with 88% versus 85%. An analogous situation was found for scholarships with a host institution in Portugal (88.9%) versus abroad (85.1%). However, it must be considered that some individuals who obtained their degree abroad may not have returned to Portugal, making it more difficult to obtain data on degree completion. The individuals aged below 35 years old at the time of application had higher success rates that their older counterparts. As far as the scientific field is concerned, the success rate was lower than the average in the Humanities and Arts and in the Social Sciences.

The percentage of scholarship recipients for whom it was impossible to determine whether or not they obtained their degree was 12.5%. This ranged from 6.9% in cohort 2 to 18.8% in cohort 4. In all cohorts, the proportion of scholars with no information/degree was higher in the Humanities and Arts than in the other fields. Out of the 726 scholarship holders for whom no information about the degree was obtained, 48 were foreigners (approximately 6.5%) and the remaining had Portuguese nationality. For 174 (24%), the degree-awarding institution was located abroad; it is reasonable to assume that they either did not obtain a doctorate or, having obtained one, did not return to Portugal. In all cohorts, scholarships awarded for studying abroad resulted in a higher percentage of recipients for whom no degree was confirmed.

**Figure 2.**
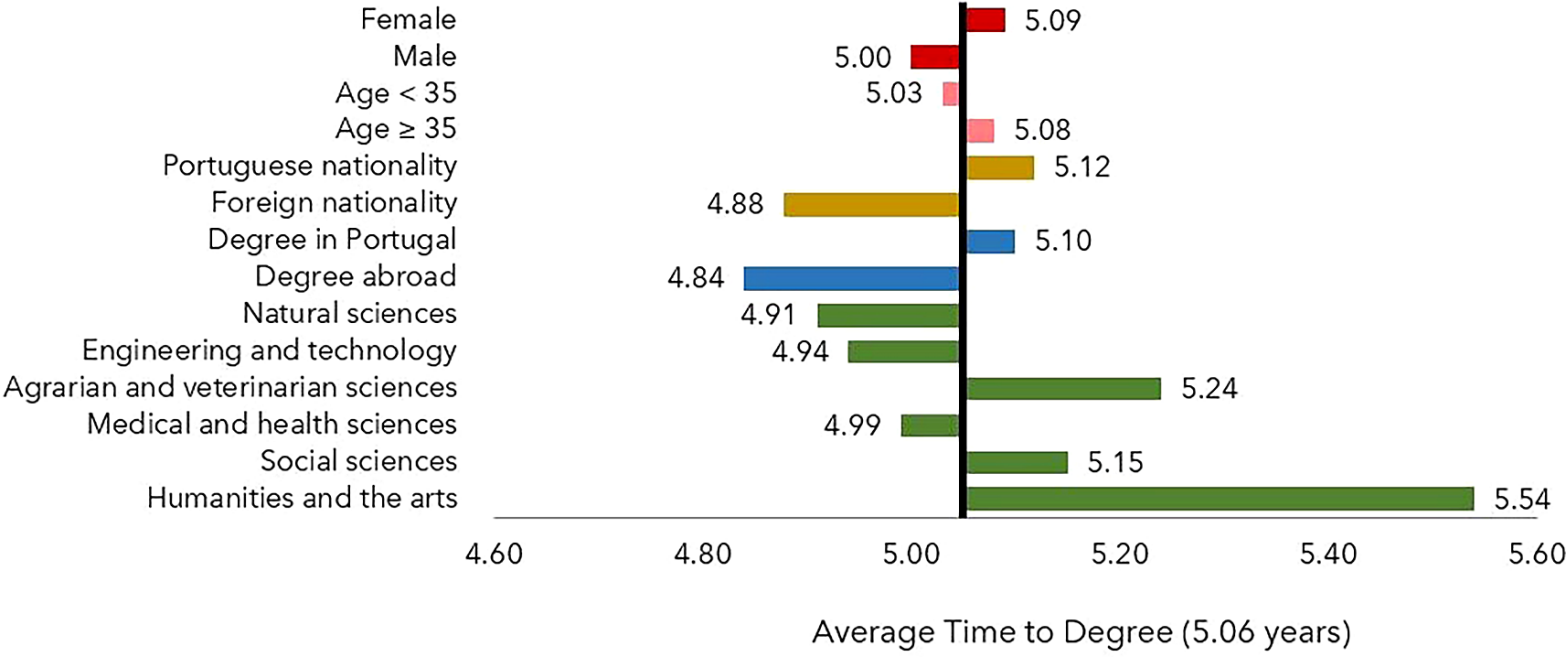
Time to degree, all cohorts. The time to degree is defined as the difference between the year of the thesis defense and the year of the beginning of the scholarship and is shown as a deviation from the average time to degree, 5.06 years. Notes: Age at application year. FORD Fields of Research & Development, OECD (2015), Frascati Manual 2015: Guidelines for Collecting and Reporting Data on Research and Experimental Development.

Neither the sex nor the age group had a statistically significant influence on the TTD ( Figure 2). In contrast, the scientific field, the nationality of the Ph.D. candidate (Portuguese or other) and the location of the degree-awarding institution (in Portugal or abroad) significantly affected the TTD, which was lower than the average for host institutions outside Portugal and for foreign scholarship holders. The TTD was found to be higher in the fields of Agrarian Sciences, Social Sciences, Humanities, and Arts compared to other scientific fields.

**Figure 3.**
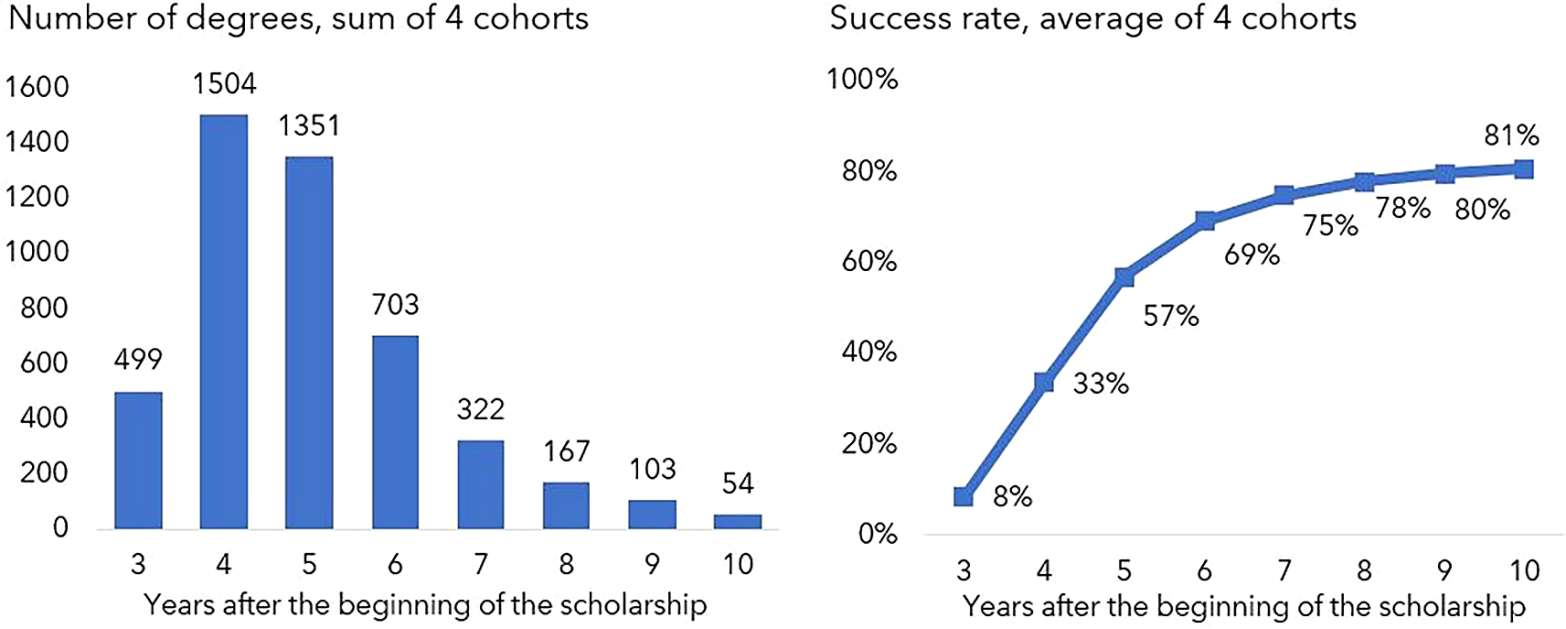
Degrees obtained 3 to 10 years after the beginning of the scholarship. The graph on the left shows the number of degrees obtained each year 3 to 10 years after the scholarship start date and the graph on the right depicts the cumulative success rate in the same period. Note: Number of individuals for whom it was possible to determine the graduation year (until July 2022).

Only 8% of the grant recipients obtained their degree within three years (the common duration of the scholarship). This figure increased to 57% within five years (see Figure 3), continued to increase significantly up to seven years (75%) and then stabilised at approximately 80% from the eighth year since the start of the scholarship. Some individuals were able to complete their degree in less than three years (not shown in Figure 3) probably because they were awarded a scholarship to complete work already funded by other sources, such as master scholarships ^3^ for older cohorts or funding from the hosting institutions for more recent cohorts.

Table 4 depicts the differences in the success rate and time to degree for each cohort and demographic characteristics of the Ph.D. candidates.

**Table 4.** Success rate and time to degree, cohort breakdown.

	Cohort							
	1		2		3		4	
	Success rate	TTD	Success rate	TTD	Success rate	TTD	Success rate	TTD
**Female**	88.9%	5.66	93.4%	5.41	90.2%	4.87	83.4%	4.76
**Male**	90.5%	5.45	92.8%	5.23	85.0%	4.85	78.1%	4.53
**Age <25**	88.8%	6.04	95.2%	5.16	89.5%	4.82	87.7%	4.66
**[25,30]**	90.2%	5.38	93.6%	5.41	90.3%	4.83	83.5%	4.65
**[30,35]**	92.3%	5.35	91.4%	5.38	86.4%	5.07	75.9%	4.59
**[35,40]**	83.3%	6.10	85.9%	5.69	79.5%	5.04	72.4%	4.76
**≥40**	81.8%	6.88	89.1%	4.75	80.5%	4.41	67.9%	5.11
**Portuguese nationality**	90.1%	5.55	93.4%	5.34	87.7%	4.89	87.7%	4.70
**Foreign nationality**	75.0%	5.33	88.9%	5.23	89.3%	4.58	89.3%	4.37
**Degree in Portugal**	92.3%	5.83	93.3%	5.44	88.4%	4.86	81.7%	4.67
**Degree abroad**	84.9%	4.94	92.7%	4.98	85.8%	4.92	77.3%	4.65
**Natural sciences**	90.0%	5.27	92.4%	5.19	92.5%	4.66	87.2%	4.49
**Engineering and technology**	90.5%	5.68	93.2%	5.11	87.3%	4.72	83.8%	4.34
**Agrarian and veterinarian sciences**	87.7%	5.68	97.9%	5.74	96.8%	5.00	91.4%	4.73
**Medical and health sciences**	91.6%	5.16	97.5%	5.41	94.3%	4.77	87.7%	4.85
**Social sciences**	88.2%	5.73	93.9%	5.44	84.0%	5.11	73.7%	4.75
**Humanities and the arts**	86.1%	6.60	88.1%	5.86	77.9%	5.26	72.6%	5.19

Success rate and time to degree for each cohort and demographic characteristics of the Ph.D. candidates.

Notes: Age at application year. TTD – Time to degree. FORD Fields of Research & Development, OECD (2015), Frascati Manual 2015: Guidelines for Collecting and Reporting Data on Research and Experimental Development.

### Gender

In cohort 1, the percentage of male scholarship holders who obtained a degree was slightly higher than that of female scholarship holders, but this was reversed in cohorts 2, 3 and 4 ( Table 4). In the last two cohorts, the success rate for female Ph.D. candidates was around 5 p.p. higher than that of males. The TTD was slightly higher for females in every cohort, but when all four cohorts are taken together, the difference between males and females is not statistically significant (M = 5.00, SD = 2.29, F = 5.09, SD = 1.95/t=-1.5, p=0.13).

### Age

As shown in TabIe 4, in all cohorts, the success rate was higher in the younger age groups. For example, in the 1995-1997 cohort, 90.2 % of scholarship holders aged [25-30] completed their degree compared to 81.8 % of those aged ≥40. In cohort 3 these percentages were 90.3% and 80.5% respectively. Although there were differences in success rates, there appears to be no correlation between the TTD of those who obtain the degree and age at the start of the scholarship. The statistical significance test (1-way ANOVA) results showed that the variances of the TTD averages between age groups were not significant (F(4.5088)=1.17, p=0.13). Therefore, the individual’s age at the start of the scholarship is not a relevant factor for TTD.

### Nationality

In cohorts 1 and 2, the completion rate of foreign scholarship holders was lower than that of Portuguese nationals ( Table 4). However, in cohorts 3 and 4, the situation changed, and foreign grant holders had a slightly higher completion rate (about 1 percentage point difference in the latter cohort). Out of the 375 foreign citizens who received funding from the FCT in all cohorts, 327 (87.2%) successfully defended their thesis. Among them, 311 completed their degree in a Portuguese institution, while the remaining 16 obtained their degree abroad but had permanent residence in Portugal. The FCT’s regulations allow for the awarding of doctoral scholarships abroad to non-nationals who meet this requirement. In all cohorts, the TTD was lower for foreign Ph.D. candidates and the difference was statistically significant (t(394)=3.98, p=8.25E-05). This is a common observation in studies published on the same subject in other countries and is often related to the fact that displaced citizens with limited funding are more motivated to complete their work and obtain a degree ( Espenshade & Rodriguez, 1997; Groenvynck et al., 2013).

### Degree in Portugal or abroad

The success rate of scholarship holders in obtaining a degree was higher when the host institution was in Portugal, except for cohort 2 where the success rates were similar for both groups ( Table 4). As stated above, caution is needed when interpreting this difference, since it is possible that some of the recipients of scholarships abroad did not return to Portugal, making it harder to ascertain the degree completion. In the first two cohorts, the time to degree was lower for those whose host institution was outside Portugal, but in cohorts 3 and 4 the difference between the two groups of scholarship holders is small. Looking at the four cohorts as a whole, the difference is statistically significant even with the levelling off of the time needed to complete doctoral training in the two most recent cohorts (t(1486)=2.54, p= 0.011).

### Scientific field and area

Across all cohorts, a lower percentage of scholarship holders in Humanities and Arts completed the doctorate compared to other scientific fields. The percentage of scholarship holders obtaining a degree was also lower in Social Sciences, except for cohort 2. Among those who obtained a doctorate, the highest TTD was found in the Humanities and Social Sciences, but also in these two fields the TTD decreased from cohort 1 to cohort 4 ( Table 4). The results of the one-way ANOVA test indicated significant differences (F(5.5087)=7.96, p=1.73E-07) in TTD among scientific fields. This finding is consistent with previous research conducted in Belgium and has been attributed to differences in academic practice ( Groenvynck et al., 2013) and Norway ( Smeby, 2000).

**Figure 4.**
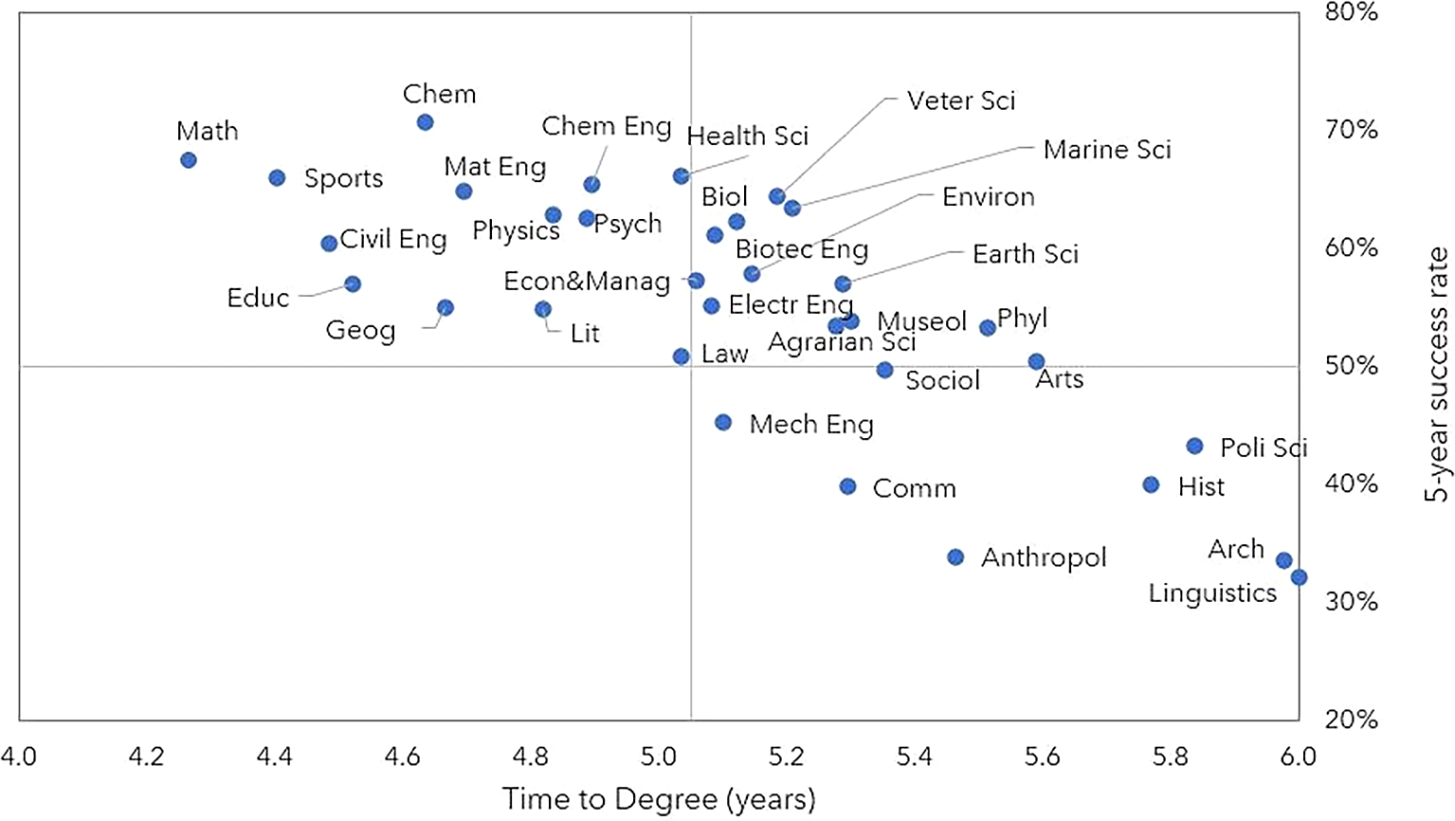
5-year success rate and time to degree per scientific area, all cohorts. Success rate 5 years after the start of the scholarship and time to degree per scientific area. Note: Number of individuals for whom it was possible to determine the graduation year (until July 2022). Anthropol = Anthropology, Arch = Architecture and Urbanism, Arts = Artistic Studies, Biol = Biology, Biotec Eng = Biotechnological Engineering, Civil Eng = Civil Engineering, Chem = Chemistry, Chem Eng = Chemical Engineering, Comm = Communication Sciences, Econ&Manag = Economics and Management, Electr Eng = Electrotechnical Engineering, Geog = Geography, Hist = History, Lit = Literary Studies, Mat Eng = Materials Engineering, Math = Mathematics, Mech Eng = Mechanical Engineering, Museol = Museology, Phyl = Phylosophy, Poli Sci = Political Sciences, Psych = Psychology, Sociol = Sociology, Veter Sci = Veterinarian Sciences.

Figure 4 shows the success rate and TTD of scientific areas. It is evident that the success rate at 5 years exceeded 50% for most scientific areas. However, there are significant variations. In the first quadrant of the graph, we find areas with a success rate over 50% but with an above-average TTD. These areas mainly include Natural Sciences and Agricultural and Veterinary Sciences (such as Biology, Marine Sciences, and Veterinary Sciences), as well as Humanities and Arts, such as Philosophy and Museology. The second quadrant identifies areas with a high 5-year success rate and below average TTD. These areas include Physics, Chemistry, and Mathematics within the field of Exact Sciences, as well as some areas of the Social Sciences (Psychology, Geography) and Humanities and Arts (Literature). Finally, the fourth quadrant (below average success at 5 years and TTD) mostly comprises areas of the Social Sciences, Humanities, and Arts, such as Linguistics, Anthropology, and History.

### Careers after the Ph.D.

**Figure 5.**
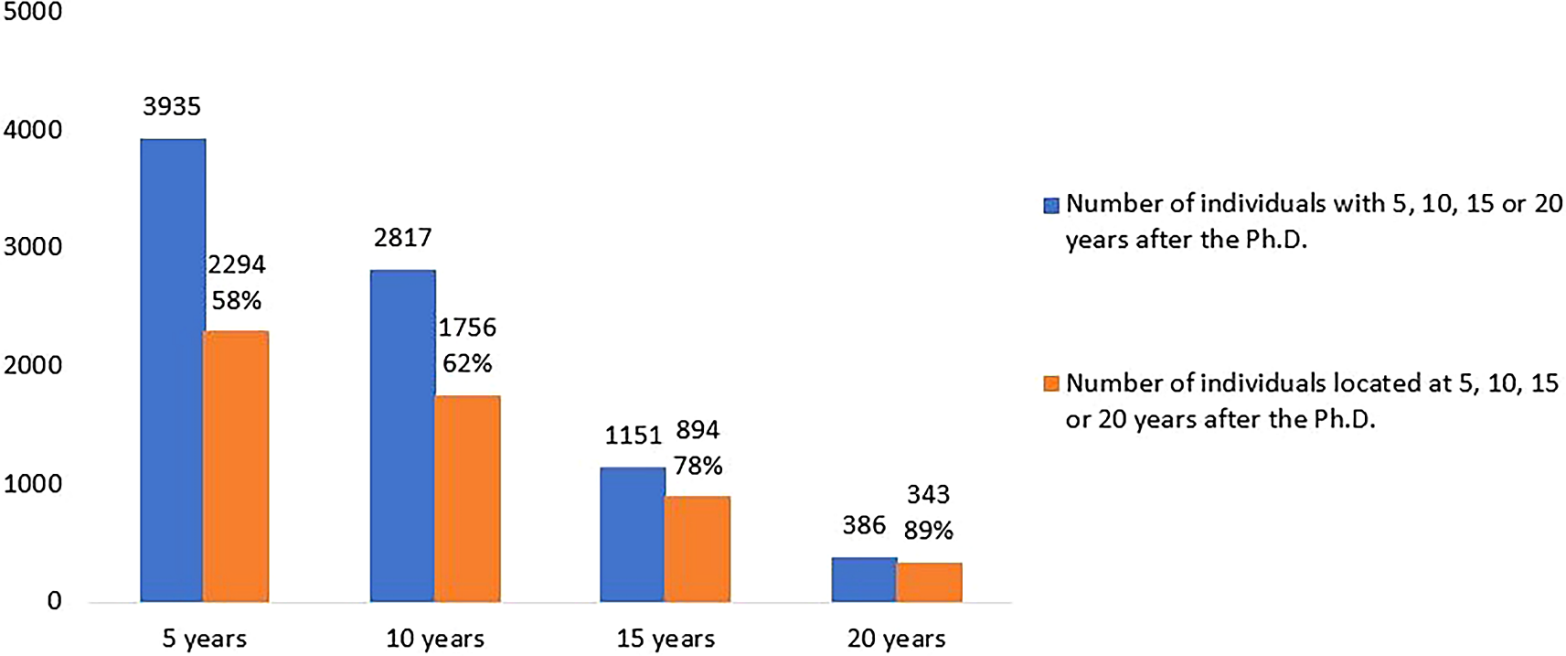
Doctorate holders located at 5, 10, 15 and 20 years after the degree, all cohorts. Number of doctoral graduates for whom 5, 10, 15 or 20 years had elapsed after the degree until 2020 (the latest year for which statistical data was available at the time of the analysis). Number and percentage of those located in Portuguese R&D institutions at each time.

Figure 5 shows the number of doctoral graduates for whom 5, 10, 15 or 20 years had elapsed after the degree until 2020 (the latest year for which statistical data was available at the time of the analysis) and the number of those located in Portuguese R&D institutions at each time. Out of the 5093 doctoral graduates analysed, 3935 had already completed at least 5 years after their doctorate. Of these, 2294 (58%) carried out R&D activity, while the remaining 42% could not be located. At the other extreme, only 386 had completed 20 years after their degree by 2020, and 343 (89%) of them were located. At 10 and 15 years after graduation, 62% and 78% of the potential Ph.D. holders were located.

**Figure 6.**
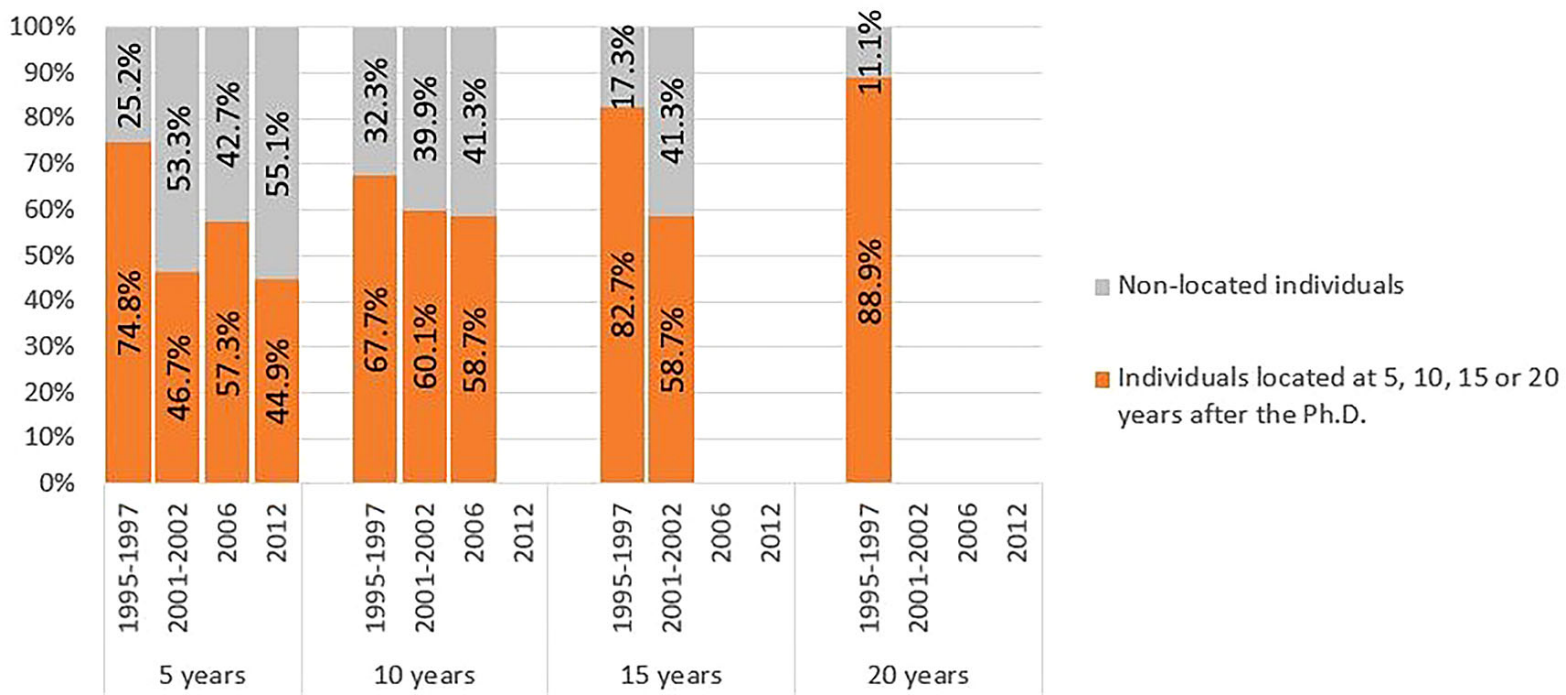
Doctorate holders located at 5, 10, 15 and 20 years after the degree, cohort breakdown. Doctoral graduates for whom 5, 10, 15 or 20 years had elapsed after the degree until 2020 (the latest year for which statistical data was available at the time of the analysis). Percentages of individuals located and non-located in Portuguese R&D institutions at each time.

When broken down by cohort and time since graduation, the proportion of located individuals tended to be higher for cohort 1 and lower for subsequent cohorts, as shown in Figure 6. The retention rate in the R&D system after 5 years was 75% for individuals in cohort 1, dropping to levels close to 50% in subsequent cohorts. In cohort 4 (scholarships started in 2012), only 156 individuals completed 5 years after graduation by 2020, and of these, only 70 were located. Due to the short follow-up period for this cohort, the results should be interpreted with caution. The persistency in R&D activity after 10 and 15 years follows a similar pattern, with a higher percentage of individuals from cohort 1 being located compared to the other cohorts. The only graduates with a follow-up time of 20 years belonged to cohort 1, with 89% still engaged in R&D.

**Figure 7.**
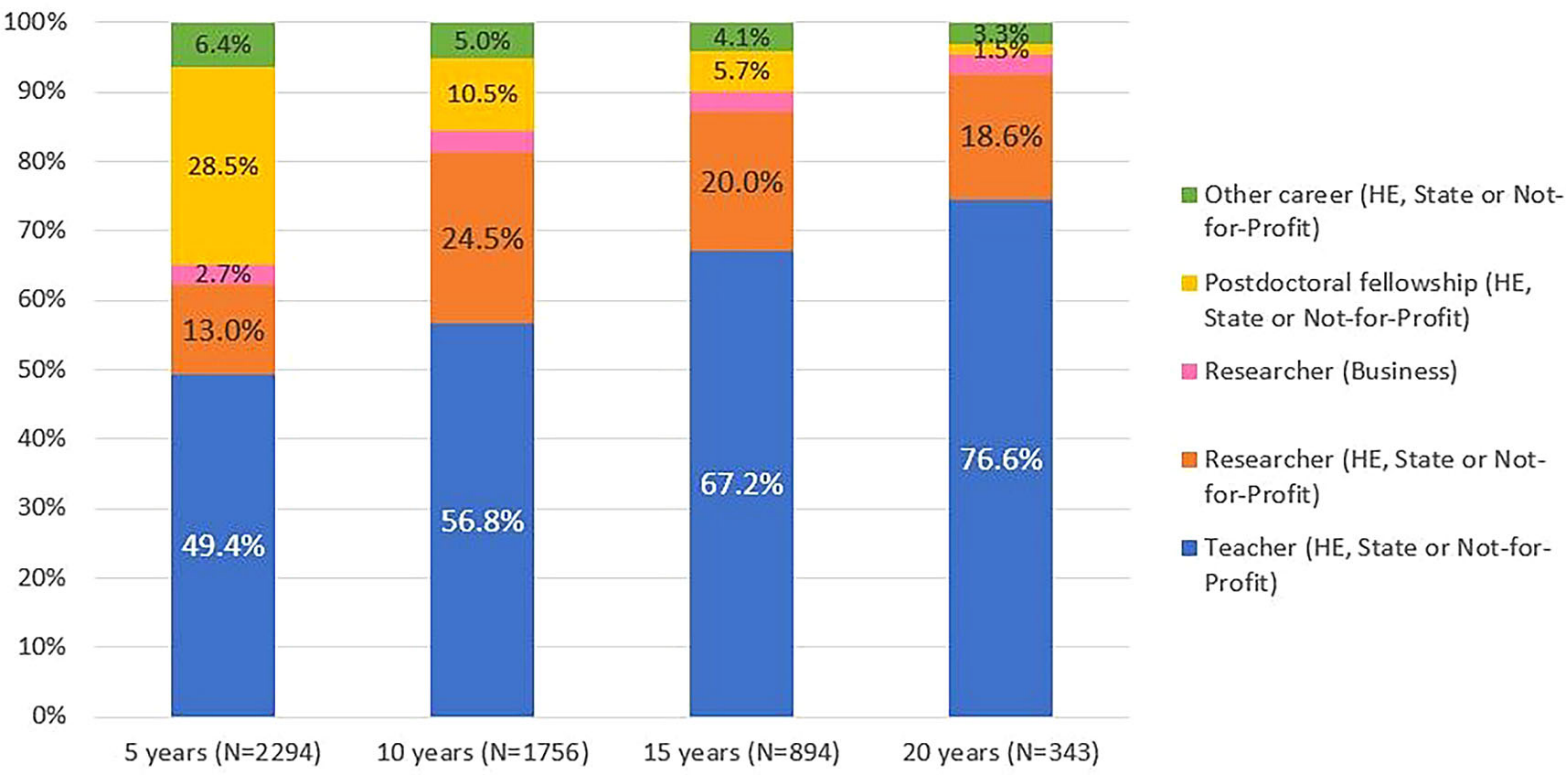
Professional situation of the doctorate holders located at 5, 10, 15 and 20 years after the degree, all cohorts. Percentual distribution of the professional situations of the doctoral graduates who were identified in the IPCTN survey at different points in time after graduation. N = number of doctoral graduates located 5, 10, 15, or 20 years after obtaining their degree (until 2020, the latest year for which statistical data was available at the time of the analysis).

Figure 7 illustrates the professional situation of the doctoral graduates who were identified in the IPCTN survey at different points in time after graduation. The information provided by the IPCTN was used to classify the professional situation as ‘ *teacher*’ (contract under the universities teaching career statutes or the polytechnic institutes teaching career statutes), ‘ *researcher*’ (contract under the research career statutes), ‘postdoctoral scholarship’ (any scholarship of varying duration) or ‘other career’. This latter situation includes medical doctors, nurses, senior health technicians, senior technicians/managers in public administration bodies, militaries or secondary education teachers who reported engagement in R&D activity.

Five years after graduation, 62% of the located graduates pursued careers in teaching or research, 29% held scholarships, and 6.4% reported other careers. Twenty years after graduation, 95% were working as teachers or researchers, with only 3.3% pursuing other careers. The proportion of individuals who reported being teachers or researchers in higher education, state or not-for-profit institutions increased with the length of time elapsed after graduation.

**Figure 8.**
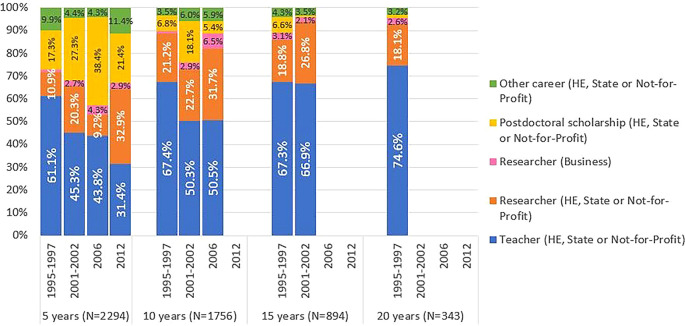
Professional situation of doctorate holders located at 5, 10, 15 and 20 years after the degree, cohort breakdown. Percentual distribution of the professional situations of the doctoral graduates who were identified in the IPCTN survey at different points in time after graduation. N = number of doctoral graduates located 5, 10, 15, or 20 years after obtaining their degree (until 2020, the latest year for which statistical data was available at the time of the analysis).

Figure 8 shows significant differences in the professional situations of graduates across different cohorts. After 5 years, 61% of the located graduates from cohort 1 were teachers, mostly in the higher education sector. This contrasts with the percentages around 45% reported by individuals of cohorts 2 and 3, where postdoctoral scholarships gained increased relevance: 27% and 38%, respectively. In fact, the role of FCT-funded postdoctoral scholarships as a means to pursue a career in R&D is evident in these cohorts. Most of the graduates from cohort 2 obtained their degrees between 2005 and 2007, during a period of significant expansion of the Portuguese R&D system. In cohort 2, 568 individuals, representing 51% of the cohort, were awarded at least one postdoctoral scholarship. In cohort 3, 31% of students obtained at least one postdoctoral scholarship. In this cohort, the majority of students completed their doctorates between 2009 and 2012. This period coincided with the impact of the 2008 financial crisis, which resulted in a significant decrease in the number of scholarships awarded by the FCT. In cohort 4, five years after graduation, there was a notable increase in the proportion of researchers employed under the Scientific Employment Stimulus Programme, which was launched in 2017 to promote the employment of Ph.D. holders and facilitate their access to research careers.

Figure 8 illustrates that the proportion of former FCT fellows pursuing careers outside of teaching and research in the business sector is consistently low across all career stages. Cohort 4 showed a slight increase at the 5-year mark since graduation. This trend can be attributed to the limited size and technological intensity of businesses in Portugal, as well as the low effectiveness of incentives to attract Ph.Ds to work outside academia.

**Figure 9.**
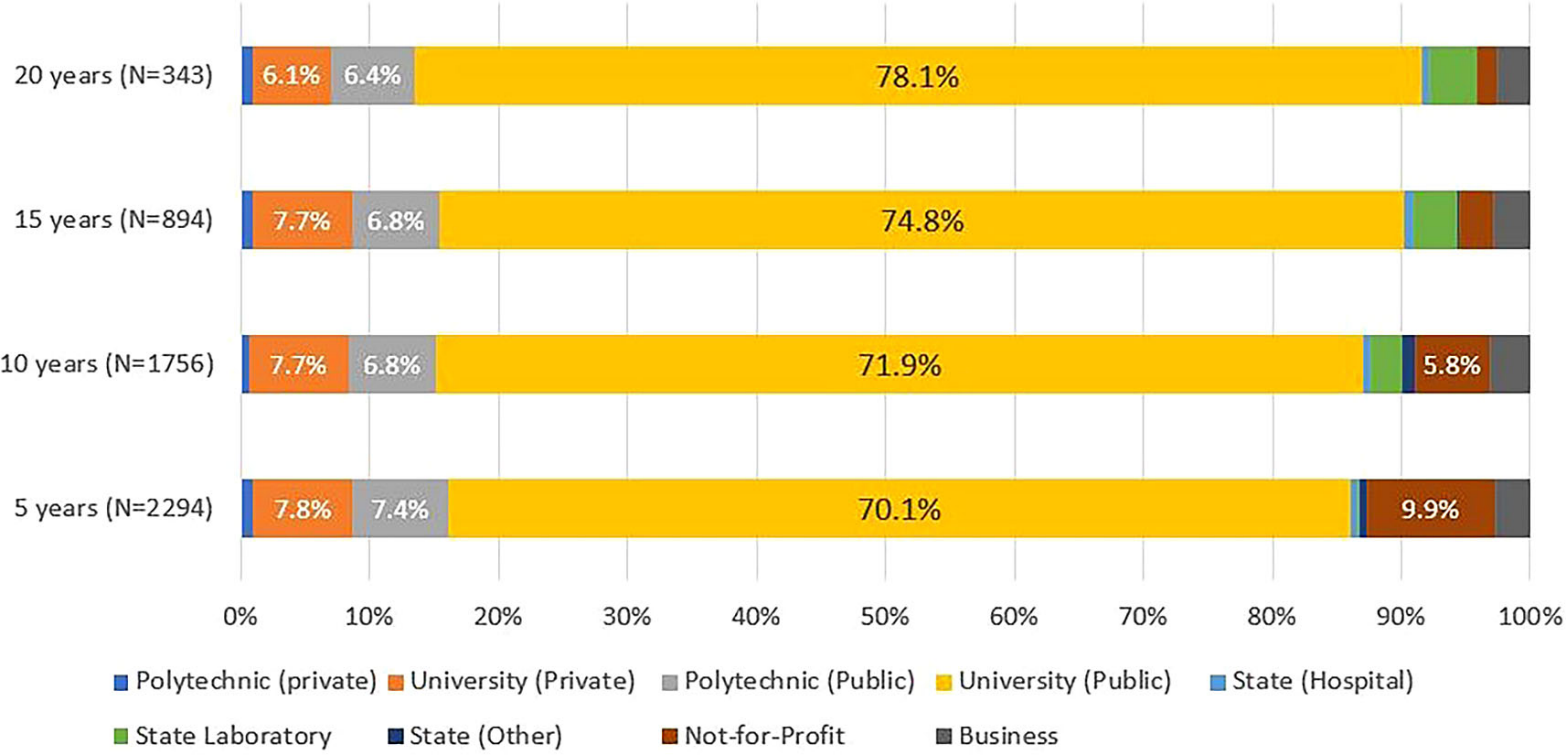
Sector of activity, all cohorts. Percentual distribution of the sectors of activity of the doctoral graduates who were identified in the IPCTN survey at different points in time after graduation. N = number of doctoral graduates located 5, 10, 15, or 20 years after obtaining their degree (until 2020, the latest year for which statistical data was available at the time of the analysis).

Around 90% of the graduates engaged in R&D activity, regardless of the number of years since their doctorate, were in higher education institutions, most often in public universities, followed by private universities and public polytechnics ( Figure 9). This was true for all cohorts and at any time since graduation.

### Snapshot of 2020

**Figure 10.**
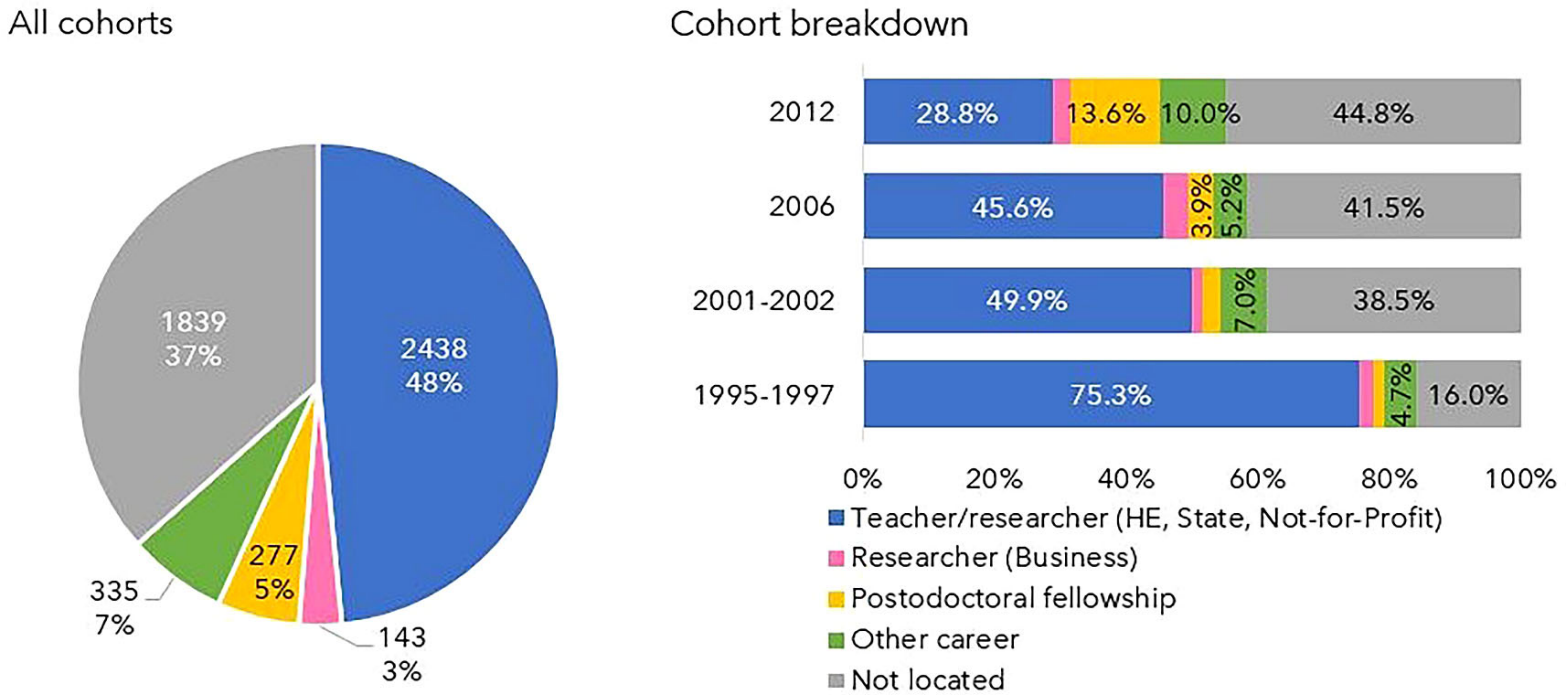
Doctorate holders located in 2020. The graph on the left shows the percentages of individuals non-located and located in Portuguese R&D institutions in 2020 and the graph on the right depicts the distribution of the professional situations of the doctoral graduates who were engaged in R&D activities.

In 2020, 3193 doctoral graduates were located out of the 5032 scholarship holders who had obtained a degree by the previous year, 2019. This corresponds to 63% of the total. The professional status of the remaining 37% could not be ascertained, as shown in Figure 10.

Almost half of the located Ph.D. holders were employed as teachers or researchers in Higher Education, the State, or Private Non-Profit Institutions. Only 5% were post-doctoral fellows, and 3% worked in R&D in the Business sector. The remaining 7% pursued other careers, which may or may not encompass R&D, such as doctors, primary/secondary school teachers, public administration managers, and military personnel.

The differences between cohorts are notable; 84% of the individuals of cohort 1 (grants started in 1995-1997) were found in 2020, a percentage that decreased steadily for the other cohorts, being 62%, 59% and 55% in cohorts 2, 3, and 4, respectively. The proportion of teachers/researchers in the state, higher education and Not-for-Profit institutions was 75% in cohort 1 versus 29% in cohort 4. This is also the cohort in which other careers and scholarships have become more representative. The proportion of researchers in the business sector did not change significantly when comparing the four cohorts and never exceeded 3.8% (cohort 3).

**Figure 11.**
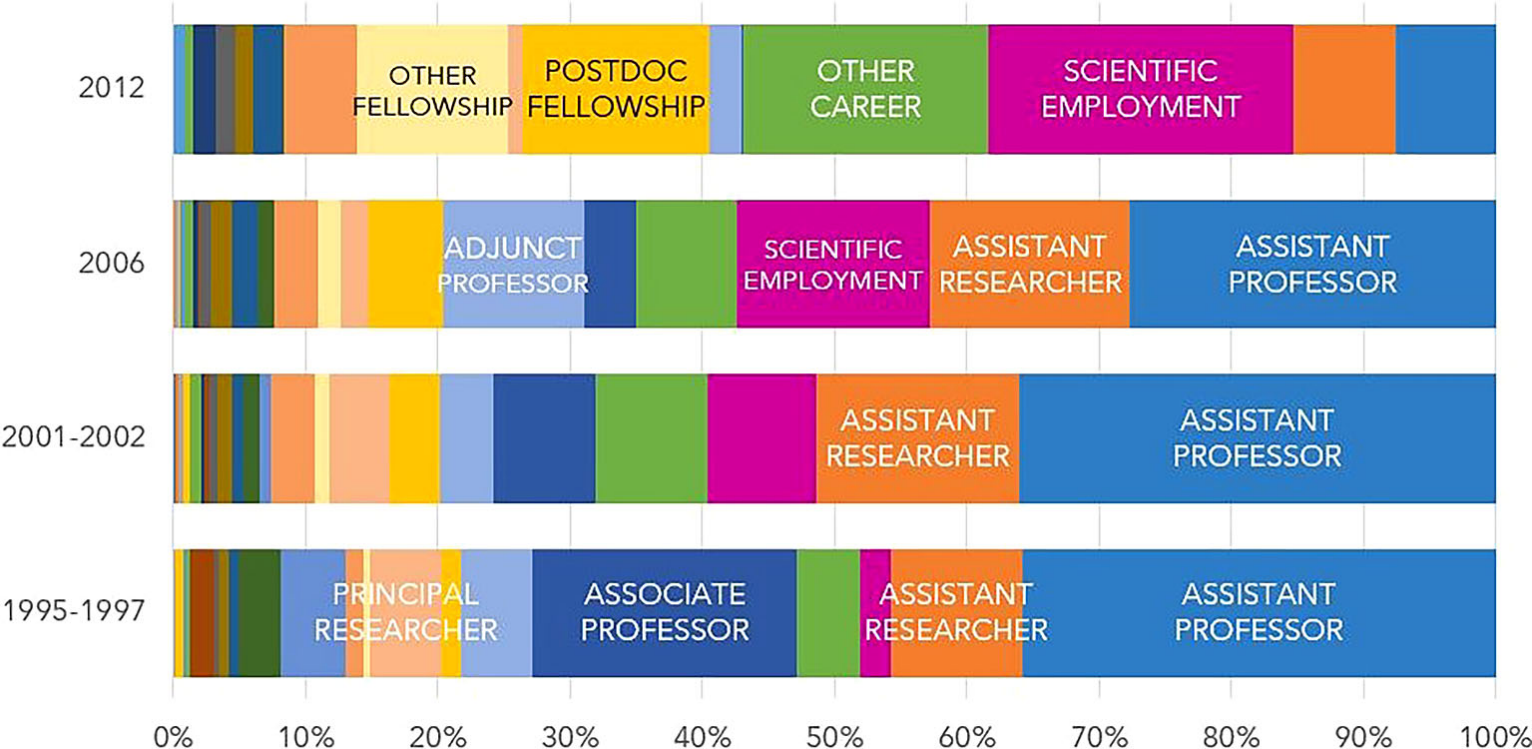
Professional situation in 2020. Percentual distribution of the professional situations/categories of the doctoral graduates who were engaged in R&D activities in 2020.

Regarding the professional status of doctoral graduates in 2020 ( Figure 11), there are noticeable differences between cohorts. The most common situation among those who started their scholarship in 2012 and obtained their doctorate mostly between 2016 and 2018 is researcher with a scientific employment contract, reported by 23% of individuals. This contrasts with the situation reported by individuals in cohort 1, who obtained their degrees between 2000 and 2003, and were primarily assistant professors/assistant researchers (46%) or associate professors/principal researchers (25%) in 2020.

It should be noted that 18.5% of the graduates from cohort 4 who had R&D activity in 2020 reported pursuing “Other career”. In contrast, only 4.7% of cohort 1 graduates were in a similar situation. The evolution from cohort 1 to 4 shows the diversification of professional contexts, since “Other career” includes senior technical or managerial positions in public administration, doctors, nurses, senior health technicians, military personnel, etc. As previously mentioned, the Scientific Employment Stimulus Programme, which began in 2016, has played a central role in the career of the individuals in cohort 4. The number of grants is also noteworthy. The ‘Other fellowships’ category comprises fellowships of several typologies and duration that can be awarded by R&D institutions to fulfill personnel needs to carry out research projects. FCT stopped awarding postdoctoral fellowships in 2016, but the data show that R&D institutions still use this mechanism and their own revenues to secure part of their R&D staff.

The proportion of former grantees with R&D activity in the business sector in 2020 was only 3% across all 4 cohorts, which is lower than the 8% estimated for all doctorate holders resident in Portugal. This suggests that former FCT fellows are less likely to be in the business sector than the general population of doctoral graduates. This issue would merit a more in-depth analysis, but it is beyond the scope of this paper.

## Discussion

### Success rate and time to degree

Identifying the factors that contribute to success in obtaining a doctoral degree can significantly inform research policy. In this study, we found that FCT funding resulted in an average success rate of 87.5%, which is comparable to that reported in similar situations, i.e. for doctorates carried out with dedicated funding over 3 to 4 years ( Groenvynck et al., 2013). The success rate was slightly higher than average for female and younger doctoral candidates, and slightly lower than average in the humanities, arts and social sciences. Studies conducted in different contexts have found little or no difference between genders in the likelihood of completing a doctorate, which is consistent with the observations in this analysis ( Mastekaasa, 2005; Ehrenberg & Mavros, 1995). On the other hand, the variation found across scientific fields supports the conclusions of previous work ( Groenvynck et al., 2013; Smeby, 2000) and has been attributed to differences in academic practice. Doctoral researchers in STEM fields (Science, Technology, Engineering and Mathematics) typically work as part of a team within the framework of a specific, pre-defined project. In contrast, their colleagues in the social sciences, arts and humanities often work independently and with less defined project parameters. These differences can provide more security, guidance, and consequently, higher success rates and shorter time-to-degree in STEM fields ( Larivière, 2012; Long & Fox, 1995).

Of those for whom it was not possible to determine whether they obtained a degree, 6.5% were foreigners. Scholarships awarded for studying abroad resulted in a higher percentage of recipients for whom no degree was confirmed across all cohorts. It is reasonable to assume that some of these individuals did not obtain a doctorate or, having succeeded, did not return to Portugal.

The consistent decrease in the time it takes to complete a degree is noteworthy. In cohort 1 (scholarships started in 1995-1997), it took 5.54 years, which decreased to 4.67 years in cohort 4 (scholarships started in 2012). This trend has also been reported in studies across different European countries ( Kyvik & Olsen, 2013; Hasgall et al., 2019). The decrease in the time to degree may be attributed to the increasing weight of doctoral programmes at the expense of the “student supervisor” model; in Portugal, the proportion of doctorates within a doctoral programme or with a “supervisory committee” was almost 60% in 2019, above the EU average and one of the highest in Europe ( EC, 2021). The improvement of doctoral training indicators was driven by additional factors, including the implementation of the Bologna Process and the introduction, in 2007, of an accreditation process that verifies if higher education institutions meet a set of conditions, such as the existence of a qualified teaching staff and adequate human and material resources, to guarantee the level and quality of training.

Several factors significantly influence the time to degree, including the scholarship holder’s nationality and the location of the host institution. Foreign scholarship holders took on average less time to obtain a doctorate than their Portuguese counterparts, as did scholarship holders who conducted their work outside Portugal. Previous publications reported similar observations, explained by the fact that expatriates with limited financial resources are more motivated to complete their work and obtain the degree ( Espenshade & Rodriguez, 1997; Groenvynck et al., 2013). The time required to obtain a degree in the Humanities, Arts, and Social Sciences was longer than in the other fields. However, as observed in the overall sample, it consistently decreased from cohort 1 to cohort 4 also in the non-STEM areas.

The results indicate positive aspects regarding the impact of the investment policies in human resources training at the doctoral level implemented by the FCT over the last 25 years. Firstly, the efficient use of public funds, including European Structural and Investment Funds, which have supported part of this investment since 1987. In fact, the success rate (or efficacy) in obtaining the degree was high and comparable to other EU. Secondly, the efficiency of scholarship recipients in obtaining a doctorate has improved over time, as evidenced by a decrease in the time taken from the oldest to the most recent cohorts. This trend is consistent with findings from similar studies.

In the more recent cohorts, fewer scholarships were awarded exclusively abroad, and more foreign students chose to pursue their Ph.D. studies in Portugal. This evolution shows the increasing maturity and attractiveness of the national R&D system. In the 1990s and early 2000s, there were significant weaknesses in scientific capacity, both in terms of human resources and investment. These limitations have been mitigated by increased R&D funding, particularly between 2005 and 2009. During this period, R&D spending grew from 0.76% of GDP to 1.58%, and the number of researchers increased from 21126 to 39834 FTE, an investment that laid the foundations for the increase in doctoral studies in Portugal.

### Careers after the Ph.D.

In the 2020 snapshot, 63% of the doctoral graduates from the four cohorts analysed were identified as having employment in higher education, where they worked as teachers, researchers or scholarship holders. The prominence of this sector aligns with official statistics regarding the overall Ph.D. population in Portugal. The cohorts exhibit significant differences, with doctoral graduates from the oldest cohort, whose fellowships began in 1995-1997 and who mostly obtained their degree by 2003, showing a remarkably high level of persistence in the national R&D ecosystem (almost 85%). In 2020, these former scholarship holders were assistant professors/assistant researchers (46%) or associate professors/principal researchers (25%). On the other hand, for the 2012 cohort, the vast majority of whom graduated in 2017, the most common situation in 2020 was a contract under the Scientific Employment Stimulus Programme, reported by 23% of individuals. The proportion of scholarships in this cohort was high, which is somewhat surprising given the policies implemented since 2016 that advocate for employment contracts as the preferred form of labour relations between doctoral graduates and R&D institutions. The number of fellowships suggests that institutions are still using this more precarious mechanism to secure their R&D workforce.

One significant observation from comparing cohorts 1 and 4 is the increasing prevalence of ‘other careers’ (6% versus 18%). This category includes senior technicians or managerial positions in public administration, doctors, nurses, senior health technicians, or military personnel in the armed forces. The 2020 snapshot indicates that FCT doctoral graduates are increasingly finding employment outside of higher education. The diversification of highly trained human resources across different sectors of society is a positive aspect and potential indicator of their growing impact. However, it is concerning that 37% of doctorate holders could not be located, raising questions about their professional status and sector of activity. It should be clarified whether they are in Portugal and what their professional status and sector of activity is. If they have left Portugal, it should be asked what they do in their host country. Answering these questions would entail the use of other research strategies, such as surveys, or additional administrative sources, such as records from the Social Security Information System.

The exercise to locate doctoral graduates at different times after their degree revealed that the proportion of individuals located was significantly higher for cohort 1 than for the other cohorts. For instance, 75% of the doctoral graduates in cohort 1 were engaged in R&D five years after their degree, a percentage that decreased to approximately 50% in cohorts 2 and 3 and reached 45% in cohort 4. After fifteen years since obtaining their PhD, 83% of cohort 1 graduates were engaged in R&D, a percentage that fell to 59% for cohort 2 graduates. This data raises questions about the ability of the national ecosystem to absorb recent doctorates. Additionally, the professional status of located doctoral graduates changed significantly with the time that elapsed after their degree and across cohorts; in cohort 1, 60% of the doctoral graduates located reported being teachers after 5 years, but in the following cohorts this percentage fell progressively, to 30% in cohort 4. Concomitantly, there was an increase in the number of researchers and post-doctoral fellows. The significance of the postdoctoral scholarships funded by the FCT as a means of ensuring continuation in the SNCT 5 years after obtaining a degree is evident in cohorts 2 and 3, while in cohort 4 the increase in researchers hired under the Scientific Employment Stimulus Programme, which has been in place since 2017, is noticeable, to the detriment of postdoctoral scholarships.

The IPCTN survey does not provide information on the type of contract, so it was not possible to determine the distribution of permanent and fixed-term contracts. However, according to the CDH survey, in 2020 65% of doctorate holders were on permanent contracts while 35% were on fixed-term contracts. The proportion of fixed-term contracts was much higher among younger doctorate holders.

Less than 20% of individuals across all cohorts, at any time since obtaining their degree, had a position outside of academia. The Higher Education sector, particularly the Public University subsector, continues to dominate overwhelmingly across all cohorts. This confirms a well-known characteristic of the national scientific ecosystem. Furthermore, the proportion of doctoral graduates in the Business sector is considerably lower than in other economies. According to the CDH, only 8% of graduates were in the Business sector, compared to 30% in countries such as Belgium, Denmark or the United States. It is worth noting some initiatives aimed at addressing this issue and capitalising on the potential of highly qualified employment in other sectors. For example, since 1997, there has been a system of tax benefits for business R&D, and since 2004, FCT doctoral scholarships are available to be carried out in companies. However, the number of applications for this type of scholarship has been quite low, typically less than 2% of the total number of scholarships awarded each year. In 2023, the FCT launched a specific application line for scholarships in non-academic environments; a total of 332 scholarships were approved. The aim was to promote and leverage doctoral programmes in the business, social, and public administration sectors. This and other measures, such as fostering scientific employment in Collaborative Laboratories ^4^, which have been in place since 2019, should contribute to reducing the imbalance between sectors in terms of the employment of doctoral graduates in the medium and long term. At the same time, other instruments, such as the “FCT Tenure” put in place in 2023, will play an important role in expanding the capacity of the scientific system to absorb doctoral graduates by providing co-funding to open-ended contracts in the teaching or research careers.

### Final remarks

This retrospective study addressed a significant gap in evaluating the return on investment in advanced human resources training by the FCT over the last 25 years. It also established the methodological foundations for monitoring the career paths of FCT-funded doctorates in the future. However, the data has some limitations that prevent a more comprehensive description of the career paths of doctoral graduates. It lacks information on international mobility and contractual relationships after graduation. Additionally, it solely relied on statistical and administrative data, omitting personal aspirations and experiences encountered during and after the doctorate. These variables are also critical in determining career paths.

The study confirmed the challenges faced by doctoral graduates funded by the FCT in integrating into non-academic job markets. This is a well-known issue in Portugal and has been identified by national and international studies. On the other hand, it has demonstrated efficient use of public funds, with high success rates and time to degree within the average of other European countries. Undoubtedly, the observed evolution between cohorts indicates the maturity attained by the Portuguese scientific system in recent decades.

## Ethics and consent

Part of the data was supplied by a third party, the Directorate-General of Statistics of Education and Science (DGEEC, https://www.dgeec.medu.pt/) whose mission is to guarantee the production and statistical analysis about education and science, upon delegation of the national body for statistics production, Statistics Portugal (INE, www.ine.pt). Access to the data was governed by a protocol signed between the FCT and the DGEEC. The authors of this paper signed a confidentiality agreement and a term of responsibility of compliance with the General Data Protection Regulation (GDPR) and with the Law of the National Statistical System, Law n° 22/2008 of 13th May 2008. Data analysis was carried out in the safe center of the Directorate-General of Statistics of Education and Science. The underlying data published in Figshare complies with the above regulations and has been treated in accordance with the international Safe Harbour Privacy Principles.

## Data Availability

The underlying data has been deposited in Figshare: Obtaining a PhD in Portugal. Determinants of success and subsequent career paths. https://doi.org/10.6084/m9.figshare.26062966 ( Ramos, A. & Ferreira, D,2024) It contains the following data:
• Time to Degree.cvs (Anonymised list of scholarship holders containing the following information: cohort number, sex, age at application year, scientific area, scientific field (ford), nationality (group), time to degree (y), degree, localinstdegree) • Professional Status After PhD.cvs (Anonymised list of scholarship holders containing the following information: cohort number, 5 years professional status, 5 years sector, 10 years professional status, 10 years sector, 15 years professional status, 15 years sector, 20 years professional status, 20 years sector) Data are available under the terms of the Creative Commons Attribution 4.0 International license (CC-BY 4.0).

## Footnotes

^1^
A permanent identifier for individuals carrying out scientific activity in Portugal, allowing authentication in various services related, such as CIÊNCIAVITAE.

^2^
CIÊNCIAVITAE is the Portuguese scientific curriculum management system.

^3^
FCT awarded master scholarships until 2007.

^4^
Collaborative Laboratories involve businesses or private non-profit associations and R&D institution to foster the production, dissemination and transmission of knowledge. Based on a portfolio of products or systems with higher added value, CoLABs aim to facilitate the access of companies to global markets through exports, as well as to support the attraction of foreign investment in technology-intensive areas.
