# Supplementary material for: Obtaining a PhD in Portugal. Determinants of Success and Subsequent Career Paths
Source: F1000Res. 2025 Feb 17;13:1101. Originally published 2024 Sep 27. [Version 2] doi: 10.12688/f1000research.150552.2 (PMC11914870; doi:10.12688/f1000research.150552.2)
Supplement: Peer review history

# Reviewer report: Reviewer response for version 2

**DOI:** 10.5256/f1000research.178007.r367037

**Published:** 2025-03-17

**Authors:** Maresi Nerad

**Affiliations:** 1 University of Washington, Seattle, Washington, USA

I have reviewed the changes made by the author and fully approve the revision to my comments. 

 I have no new comments to make. Good work

 Maresi Nerad

Is the work clearly and accurately presented and does it cite the current literature?

Partly

If applicable, is the statistical analysis and its interpretation appropriate?

Yes

Are all the source data underlying the results available to ensure full reproducibility?

Yes

Is the study design appropriate and is the work technically sound?

Yes

Are the conclusions drawn adequately supported by the results?

Partly

Are sufficient details of methods and analysis provided to allow replication by others?

Yes

Reviewer Expertise:

Doctoral education research and policies, PhD career path studies,  globalization of doctoral education, the history of women in higher education

I confirm that I have read this submission and believe that I have an appropriate level of expertise to confirm that it is of an acceptable scientific standard.

# Reviewer report: Reviewer response for version 2

**DOI:** 10.5256/f1000research.178007.r367038

**Published:** 2025-02-26

**Authors:** Sally Hancock

**Affiliations:** 1 University of York, York, England, UK

Thank you for the opportunity to read a revised version of this manuscript. In my view the authors have responded comprehensively to the peer review feedback and the paper is strengthened as a result.

Is the work clearly and accurately presented and does it cite the current literature?

Yes

If applicable, is the statistical analysis and its interpretation appropriate?

Yes

Are all the source data underlying the results available to ensure full reproducibility?

Yes

Is the study design appropriate and is the work technically sound?

Yes

Are the conclusions drawn adequately supported by the results?

Partly

Are sufficient details of methods and analysis provided to allow replication by others?

Yes

Reviewer Expertise:

Doctoral education, doctoral employment, higher education policy

I confirm that I have read this submission and believe that I have an appropriate level of expertise to confirm that it is of an acceptable scientific standard.

# Reviewer report: Reviewer response for version 1

**DOI:** 10.5256/f1000research.165133.r347736

**Published:** 2025-01-15

**Authors:** Maresi Nerad

**Affiliations:** 1 University of Washington, Seattle, Washington, USA

This study is a useful analysis of doctoral completion rate, time-to-degree, and careers at 5, 10, 15 and 20 years out of 4 cohorts of Portuguese PhD fellowship holders funded by the Portuguese Foundation for Science and Technology between 1995 and 2012.  It is useful as there seem to be a lack of such analyses of fellowship holders that can be compared to European data on PhDs. Specifically, the funding agency is finding out whether their monetary investment made a difference in completion rates and time-to-degree for PhDs and whether the PhDs joined R&D organizations, be there in business, industry, government of academia.

 The article is well written and mostly clear. The data sources are well defined, and the method is clearly described. The findings are well supported, and the supplied tables and description are transparent. The conclusion drawn to answer the 3 ^rd^ research question, particularly in terms of relevance for public policy could be more concise. The authors correctly mention three important limitations of the study.  There are contradictions in the interpretation of the career findings.

 The following suggestions and recommendations follow the sequence of the article:

 Introduction and literature review

 To make it easier for non-Portuguese readers I would recommend providing at the beginning of the article explanations of the specific Portuguese context or refer in the introduction to the pages in the article where various new Portuguese policies are described.

 The literature review could be improved by consulting a wider range of literature on PhD career paths, especially the US National Science Foundation reports (Survey of Doctoral Recipients – SDR biannual report, and the Survey of Earned Doctorates, SED annual report), both are analyzed, and data is provided in the NSF *Science & Engineering Indicators* Publication. Such studies have been going on since 1950s.  Already in 1999 *Science *published an article on a national US PhD career path study, explaining the different patterns of choosing to undertake postdoc studies by selected science and engineering disciplines, Postdoctoral Patterns, Career Advancement, and Problems, in American Association for the Advancement of Science, *Science*, 285, Vol 285, pp.1533-1535). This article explained that gender needs to be carefully considered.  The Center for Innovation and Research at the University of Washing, Seattle has further undertaken 2 more US national career path studies and advised European organizations on how to track researchers’ career. See for example in 2012 a meeting in Luxembourg under the leadership of the European Science Foundation  ( https://www.education.uw.edu/cirge/category/phd-career-path-tracking/).

 The authors mention a publication by Ehrenberg and Mavros (1995). It is suggested to look further into Ehrenberg’s publications, such as the 2009 book Ehrenberg & Kuh (Eds) *Doctoral Education and the Faculty of the Future, *Ithaka, NY: Cornell University Press.) See also the reports from the US Council of Graduate Schools (CGS) on studies on doctoral completion.  Similarly, the UK Council on Graduate Education (UKCGE) has published various PhD career path studies. Also note the Australian study on PhDs career path study of 2007 (Western et al. PhD Graduates 5 to 7 Years Out: Employment Outcomes, Job Attributes and the Quality of Research Training, Brisbane, Australia: The University of Queensland Social Research Center).

 The authors write that “There is currently limited understanding of the expansion of doctoral training, including its causes, social and economic contributions, and medium to long-term.”

 It is advisable that the authors familiarize themselves better with the publication in the field of higher education, particularly doctoral education research.  For example, Portuguese colleagues (Cardoso, et al) published a book in 2020, *Structural and Institutional Transformations in Doctoral Education, *Issues in Higher Education *) *that has several chapters explaining the expansion in doctoral education worldwide.

 Methods

 National career path studies solely relying on administrative and nationally collective data, are an intricate undertaken. The characterization of the cohorts and thus the analysis of the studies’ findings would benefit from disaggregating men and women by disciplines and age. For example, more women are fellowship holders among cohort 3 and 4, and in these cohorts the percentage of social sciences, humanities and arts has increases. Traditionally women tend to study to a larger degree in these fields, which could be an explanation of the increase of in the last two cohorts.  

 The cohorts are well described. It would be good to include the rationale for the grouping of sample sizes of the 4 cohorts (three years of graduates in the first cohort, two years in the second cohort, and one year in the fourth cohort). Once the readers see the numbers in the tables, they understand that the authors opted for an even cohort population. 

 Results and Discussion

 The authors name the limitation of the study: lacking information on international mobility in the career, the kind of contractual appointment such a temporary or more permanent appointments, part time or full-time, and most importantly, the lack of information of personal career aspiration. However, further contextual information, such as being a parent and needing to provide income for the family, might restrict some newly completed PhDs to accept temporary postdoc positions in favor of other more permanent employment options.

 The study’s research questions 1 and 2 are well answered with clear evidence in the data findings.  The authors could be more explicit in answering the third study question in providing conclusions and possible recommendations that are relevant for public policies and that might lead to an increase of PhDs choosing R&D employment both within academia and in business and industry.

 Minor suggestions

 The term used for non-Portuguese citizens who have permanent residence in Portugal as “displaced citizen”, is not a commonly used term.  

 It would be good to define PhD “success”. What is considered a successful time for completing a PhD in Portugal?

 What is not mentioned in the article is the importance of the quality of the outcome, i.e. the trained doctorate as well as the quality of doctoral researcher for labour market analyses.  In this study quality could not be assessed by using the existing data sources.

 On page 17, could the underlying assumption in this sentence be explained?

 “The number of fellowships suggests that institutions are still using this more precarious mechanism to secure their R&D workforce.”  

 A follow up study comparing fellowship holders with non-fellowship holders might be interesting and might provide another validation of the investment of money by the FCT in doctoral fellowships.

Is the work clearly and accurately presented and does it cite the current literature?

Partly

If applicable, is the statistical analysis and its interpretation appropriate?

Yes

Are all the source data underlying the results available to ensure full reproducibility?

Yes

Is the study design appropriate and is the work technically sound?

Yes

Are the conclusions drawn adequately supported by the results?

Partly

Are sufficient details of methods and analysis provided to allow replication by others?

Yes

Reviewer Expertise:

Doctoral education research and policies, PhD career path studies,  globalization of doctoral education, the history of women in higher education

I confirm that I have read this submission and believe that I have an appropriate level of expertise to confirm that it is of an acceptable scientific standard, however I have significant reservations, as outlined above.

# Response

**Published:** 2025-02-06

**Authors:** Ana Ramos

**Affiliations:** Studies and Planning, Fundacao para a Ciencia e a Tecnologia, Lisbon, Lisbon, Portugal

The authors would like to express their gratitude to Professor Maresi Nerad for her meticulous analysis of the manuscript and her constructive feedback. In fact, the literature review concerning the career trajectories of doctorate holders was not sufficiently comprehensive. Therefore, information regarding the situations found in the surveys carried out in the USA and Australia was incorporated. Additionally, a reference to a recent study focused on doctoral education in Portugal was included; this study does not deal directly with completion rates and subsequent trajectories, but it is very useful to contextualise the Portuguese situation.

 As suggested, a phrase has been inserted at the end of the Introduction, in the section entitled 'Doctoral training in Portugal – background', to inform the reader that further information about the Portuguese context and policies can be found in the subsequent sections (Page 3, Line 32).

**Main changes**

**Introduction - Career paths of doctoral graduates**

**The sentence:** *There is currently limited understanding of the expansion of doctoral training, including its causes, social and economic contributions, and medium to long-term consequences*

**Was changed to (Page 3, Lines 34-36)**

*A more comprehensive understanding of the expansion of doctoral training, including its causes, social and economic contributions, and medium to long-term consequences is needed.*

**The following text was added (Page 3, Lines 40-45)**

*In the USA, information about doctoral careers is consistently gathered; the National Science Foundation sponsors two surveys on doctoral education, the Survey of Earned Doctorates (a census of all doctoral graduates, since 1957) and the Survey of Doctorate Recipients (a biennial survey tracking PhD holders in science, engineering, and health with its first edition in 1973). Also, the Council of Graduate Schools collects data through its PhD Career Pathways Project.*

**A new phrase mentioning the EC/OECD observatory of R&I careers (ReICO) was introduced (Page 4, Lines 4-7)**

*Furthermore, the Research and Innovation Careers Observatory (ReICO), launched in 2024 by the European Commission and the OECD with the aim to develop a repository for monitoring trends in research and innovation careers, will constitute a valuable source for future studies.*

**Information about doctoral graduates in the US and Australia was included (Page 4, Lines 23-32)**

*In the US, more than 50% of doctorates in science, engineering, and health were employed in professions outside of academia within 10-14 years of graduating (as was the case in the last 20 years). Moreover, over 90% of respondents report job satisfaction 15+ years after getting their doctorate. It was also shown that most recent doctoral graduates engage in research and development, regardless of employment sector (National Science Board, National Science Foundation, 2024). In Australia, circa 50% of PhD graduates were employed outside academia, in sectors such as research and development, government, private industry, healthcare, and non-profits and many had moved away from their doctoral-granting institutions, indicating a high degree of geographical mobility in the pursuit of career opportunities (Western et al. 2007).*

**Contextual information about doctoral education in Portugal was added (Page 5, Lines 8-14)**

*The Portuguese doctoral education landscape was the subject of a recent study by Cardoso et al (2024), which examined the evolving nature of doctoral education in Portugal. The study demonstrated that Portuguese universities are adapting to broader European trends in doctoral education, balancing the need for rigorous academic research with the demand for transferable skills applicable beyond academia. The study provides valuable insights into how doctoral education in Portugal is evolving to meet contemporary academic and professional challenges.*

**The following additional bibliographic references were incorporated:**

- National Science Board, National Science Foundation. 2024. *Science and Engineering Indicators 2024:* *The State of U.S. Science and Engineering*. NSB-2024-3. Alexandria, VA. Available at https://ncses.nsf.gov/pubs/nsb20243.
- Western, M, Boreham, P, Kubler, M, Laffan, W, Western, J, Lawson, A & Clague, D 2007, PhD graduates 5 to 7 years out: employment outcomes, job attributes and the quality of research training: final report (revised), The University of Queensland (Brisbane, QLD).
- Cardoso, S., Amaral, A., & Carvalho, T. (2024). The changing concept of doctoral education: an insight from Portuguese universities. *Journal of Further and Higher Education*, *48*(2), 255–268. https://doi.org/10.1080/0309877X.2024.2302035

**Methods**

 Professor Merad suggests that “the characterization of the cohorts and thus the analysis of the studies’ findings would benefit from disaggregating men and women by disciplines and age”.

 We agree that this disaggregation would undoubtedly yield valuable insights into the evolution and status of men and women in terms of PhD completion and time to degree (by discipline and age). However, this would fall outside the primary objective and scope of the present work, which was to provide an initial characterisation of recipients of doctoral scholarships and, for the first time, conclude on the return of this investment of the FCT, which funds science and technology across all scientific domains. We are aware that a more detailed analysis of the data would enrich the study, its conclusions and its implications for policy. In fact, the FCT and the authors of the present paper are implementing a monitoring mechanism of all doctoral scholarship recipients (success, time to degree and professional trajectories). The goal is to provide a comprehensive picture, through reports, international publications and interactive dashboards that will enable the visualisation of disaggregated indicators by sex, age, and scientific discipline.

**Comment: ** “Include the rationale for the grouping of sample sizes of the 4 cohorts (three years of graduates in the first cohort, two years in the second cohort, and one year in the fourth cohort)”. 

**Author Response: **A phrase was inserted to clarify this rationale, as suggested (Page 6, Lines 7-9)

**Results and Discussion**

**Comment: **The authors could be more explicit in answering the third study question in providing conclusions and possible recommendations that are relevant for public policies and that might lead to an increase of PhDs choosing R&D employment both within academia and in business and industry.

**Author Response: **A new paragraph was added to the “Final remarks” subsection to accommodate this comment and establish a connection between our results and relevant public policies for fostering attractive careers in academia and in other sectors (Page 23)

**Comment: Minor suggestions:**

 The term used for non-Portuguese citizens who have permanent residence in Portugal as “displaced citizen”, is not a commonly used term.  

**Author Response: **The term “displaced” was changed to “expatriates” (Page 13, Line 29).

**Comment: **It would be good to define PhD “success”. What is considered a successful time for completing a PhD in Portugal?

**Author Response: **Throughout the paper, “success” is defined as the individual having obtained the Ph.D. until the time of analysis (July 2022), regardless of the time elapsed since the beginning of the scholarship (this is stated in the Note of Figure 1). For the sake of clarity, this information was added to the sentence in Page 7, Lines 1-2.

**Comment: **What is not mentioned in the article is the importance of the quality of the outcome, i.e. the trained doctorate as well as the quality of doctoral researcher for labour market analyses. In this study quality could not be assessed by using the existing data sources.

**Author Response: **The reviewer is correct; this qualitative assessment would have to be done resorting to additional experimental approaches.

**Comment: **On page 17, could the underlying assumption in this sentence be explained?

 “The number of fellowships suggests that institutions are still using this more precarious mechanism to secure their R&D workforce.”

**Author Response: **The phrase was rewritten and amended to provide an explanation/rationale (Page 21, Lines 29-31).

**Comment: **A follow up study comparing fellowship holders with non-fellowship holders might be interesting and might provide another validation of the investment of money by the FCT in doctoral fellowships.

**Author Response: **Contrafactual analysis is indeed a powerful tool and a study to this effect is planned for 2025 as part of the Portuguese obligation to evaluate ex post the impact of European Structural and Cohesion Funds (which are used to partially finance doctoral fellowships in Portugal). The FCT will be involved in this contrafactual analysis, which will contribute to elucidate aspects that could not be tackled in the present study.

# Reviewer report: Reviewer response for version 1

**DOI:** 10.5256/f1000research.165133.r334906

**Published:** 2024-11-13

**Authors:** Sally Hancock

**Affiliations:** 1 University of York, York, England, UK

This article offers an important contribution to our understanding of the changing employment conditions for PhD holders in age of expansion. It does so through analysis of an extensive dataset on PhD holders in Portugal and is able to highlight shifts in employment destinations over time. The unique case of doctoral expansion in Portugal is well explained and the article is well positioned in relation to current literature. The study design and presentation of results are clear. The analysis appears to be sound. The most significant aspect for further development relates to the interpretation of the empirical data and concluding discussion. As a reviewer I remain somewhat undecided about the strength of the negative framing of the trend away from academic and R&D employment. Does this implicitly convey policy-makers' and employers' aspirations for growing the doctoral supply chain, and if so, should that aim be more explicit? Since the survey data are descriptive and do not robustly convey individual decision-making, it is not possible to say whether this shift is a disappointment for the PhD holders concerned, or whether it represents a broadening of horizons for action and a deliberate venture into non-academic employment. It seems, to me, unwarranted to valorise the empirical observations by referring to 'difficulties in retaining' PhDs in academic or research employment, or of the 'challenges of integrating' PhDs in the non-academic labour market. These trends may be an inevitable phase in the ongoing expansion of doctoral labour market and may lead to transformations in non-academic sectors. I think there is a wider point to acknowledge, which is that the contributions of PhD graduates beyond academic and research roles are not well understood, but that does not necessarily mean that value is not created here. Also, with regard to the way the trends over time are framed: the declining 'persistence' in R&D employment across cohorts may not necessarily be a cause for concern. It may be a purposeful turn away from precarious and deteriorating academic working conditions made by younger PhD holders. Finally, it may be worth some comment on the fact that although the data can show trends over time, they cannot capture individual trajectories. It is not clear if these trends will have permanence or if there may be individual movement between academic, R&D and non-academic occupations over the career (sector porosity) - meaning that some of these destinations are less static than might be implied.

Is the work clearly and accurately presented and does it cite the current literature?

Yes

If applicable, is the statistical analysis and its interpretation appropriate?

Yes

Are all the source data underlying the results available to ensure full reproducibility?

Yes

Is the study design appropriate and is the work technically sound?

Yes

Are the conclusions drawn adequately supported by the results?

Partly

Are sufficient details of methods and analysis provided to allow replication by others?

Yes

Reviewer Expertise:

Doctoral education, doctoral employment, higher education policy

I confirm that I have read this submission and believe that I have an appropriate level of expertise to confirm that it is of an acceptable scientific standard, however I have significant reservations, as outlined above.

# Response

**Published:** 2025-02-06

**Authors:** Ana Ramos

**Affiliations:** Studies and Planning, Fundacao para a Ciencia e a Tecnologia, Lisbon, Lisbon, Portugal

The authors would like to thank Dr. Sally Hancock for her careful reading of the manuscript and her constructive comments. We agree that probably there was an excessive "negative framing" of the decreased persistence of graduates in the academy from cohort 1 to cohort 4. Indeed, this trend may reflect individual deliberate choices for increasingly diversified careers and/or a shift away from highly precarious academic positions. We have therefore made several changes (please see the revised version with track changes) that mitigate the negative framing of the observation, acknowledging that the reasons for such reduced persistence are multifactorial, could not be fully captured given the limitations of the study, and are not necessarily negative for the evolution of the Portuguese science system. Changes were made in:

**Abstract (Page 2)**

 The following sentence was removed: “suggesting difficulties in retaining recent graduates in academia and in the private sector”

 The sentence: *This study confirmed the challenges faced by doctoral graduates in integrating into non-academic job markets w*as changed to:

*This study showed that there are still challenges to address concerning the integration of doctoral graduates into non-academic research positions*

**Discussion – careers after the Ph.D.**

 The sentence *However, it is concerning that 37% of doctorate holders could not be located*, raising questions about their professional status and sector of activity” was changed to

*However, 37% of doctorate holders could not be located, raising questions about their professional status and sector of activity* (Page 21, Line 38)

 The following sentence was added (Page 21, Lines 44-46 and Page 22, Lines 1-3):

*It is not possible to ascertain whether the shift from research to non-research careers is mostly due to lack of opportunities in the former sector or to a strategic move of graduates to other sectors of employment and increasingly diversified careers that ultimately can lead to a desirable societal and economic transformation of the country. Further studies would be necessary to shed light on the individual motivations of recent Ph.D. graduates, as well as about intersectoral mobility, that could not be adequately captured in the present study.*

 The following sentence was removed (page 22, Lines 9-10):

*This data raises questions about the ability of the national ecosystem to absorb recent doctorates*

**Final remarks**

 The second paragraph now reads as follows (Page 23, Lines 10-19):

*There are still challenges to be addressed to foster attractive careers for Ph.D. holders, both within and beyond academia. Key factors in this regard include the reduction of the precarious nature of academic positions, the establishment of a framework conducive to diversified career tracks and the promotion of international and intersectoral mobility. In recent years, policies towards these goals have been put in place, but it is evident that an increase of the public R&D investment is needed for achieving the objectives. In the business sector, it is imperative to enhance the recognition of the added value of a Ph.D. graduate to improve the performance and the internationalisation ability of enterprises. FCT-funded doctoral scholarships in non-academic contexts rose from 22% in 2023 to 30% in 2024; in the medium to long term, this change is expected to impact careers outside academia.*
